# Comprehensive Real-Time RT-PCR Assays for the Detection of Fifteen Viruses Infecting *Prunus* spp.

**DOI:** 10.3390/plants9020273

**Published:** 2020-02-19

**Authors:** Alfredo Diaz-Lara, Kristian Stevens, Vicki Klaassen, Deborah Golino, Maher Al Rwahnih

**Affiliations:** 1 Department of Plant Pathology, University of California-Davis, Davis, CA 95616, USA; adiazlara@ucdavis.edu (A.D.-L.); dagolino@ucdavis.edu (D.G.); 2 Department of Evolution and Ecology, University of California-Davis, Davis, CA 95616, USA; kastevens@ucdavis.edu; 3 Foundation Plant Services, University of California-Davis, Davis, CA 95616, USA; vaklaassen@ucdavis.edu

**Keywords:** *Prunus* spp., viruses, genetic diversity, detection, real-time RT-PCR, high throughput sequencing

## Abstract

Viruses can cause economic losses in fruit trees, including *Prunus* spp., by reducing yield and marketable fruit. Given the genetic diversity of viruses, reliable diagnostic methods relying on PCR are critical in determining viral infection in fruit trees. This study evaluated the broad-range detection capacity of currently available real-time RT-PCR assays for *Prunus*-infecting viruses and developed new assays when current tests were inadequate or absent. Available assays for 15 different viruses were exhaustively evaluated in silico to determine their capacity to detect virus isolates deposited in GenBank. During this evaluation, several isolates deposited since the assay was designed exhibited nucleotide mismatches in relation to the existing assay’s primer sequences. In cases where updating an existing assay was impractical, we performed a redesign with the dual goals of assay compactness and comprehensive inclusion of genetic diversity. The efficiency of each developed assay was determined by a standard curve. To validate the assay designs, we tested them against a comprehensive set of 87 positive and negative *Prunus* samples independently analyzed by high throughput sequencing. As a result, all the real-time RT-PCR assays described herein successfully detected the different viruses and their corresponding isolates. To further validate the new and updated assays a *Prunus* germplasm collection was surveyed. The sensitive and reliable detection methods described here will be used for the large-scale pathogen testing required to maintain the highest quality nursery stock.

## 1. Introduction

Fruit trees are grown worldwide, mainly as a food source, and *Prunus* spp. are one of the most popular cultivated trees. The genus *Prunus* includes almonds, apricots, cherries, peaches, plums, and nectarines. In the United States, the main producers of *Prunus* spp. are the states of California, Washington, Oregon, South Carolina, Georgia, Michigan, and New Jersey (https://www.nass.usda.gov/index.php).

Although economic losses due to viral infection in *Prunus* spp. are difficult to quantify, viruses can cause losses by reducing plant vigor and growth, delaying fruit ripening, and causing graft and compatibility issues. Viruses can also remain latent, later, causing plants to grow slowly, produce smaller fruit, and have a reduced lifespan, but often these detrimental impacts may go unnoticed unless crops are visibly damaged [1]. Reduction in yield and poor product quality from some viruses can be severe and lead to tree removal. Some major viruses of *Prunus* spp. include apple chlorotic leaf spot virus (ACLSV), cherry green ring mottle virus (CGRMV), cherry leaf roll virus (CLRV), little cherry virus-1 and -2 (LChV-1 and -2), prune dwarf virus (PDV), and Prunus necrotic ringspot virus (PNRSV). Cemballi et al. [2] estimated that the United States sweet cherry and clingstone peach industries could save $11,191,460 and $5,580,877, respectively, adopting a virus protection program.

The genetic diversity of plant viruses is well known (reviewed in [3,4]). For example, divergent variants of LChV-1 and LChV-2 have been characterized via high throughput sequencing (HTS), which affects the epidemiology and symptomatology associated with these viruses [5,6]. PNRSV and PDV isolates can be classified in several phylogroups based on their coat protein (CP) or RNA-dependent RNA polymerase (RdRp) genes [7,8]. This genetic diversity makes it difficult to design and maintain sensitive assays that will reliably detect different virus isolates. Nucleic acid specific detection of viruses is a very useful technique to determine if a particular virus is present in a plant. Methods relying on PCR require sequence specific primers which may not amplify a virus sequence if the virus has nucleotide differences at the primer binding site. Given the genetic diversity of viruses, it is possible to miss detection of virus strains if the PCR assay is not specific for a variant present in nature.

Efficient and reliable laboratory diagnostic tests are critical in determining viral infection in *Prunus* spp. While several diagnostic methods are available for viral detection (e.g., biological indexing and ELISA), the advantages of using real-time reverse transcription PCR (RT-PCR) to detect viruses have been documented. The development of real-time RT-PCR assays led to superior sensitivity, speed, reproducibility, and limited risk of contamination compared to end-point RT-PCR [9,10]. The main feature of real-time RT-PCR is that DNA amplification is detected in real time as RT-PCR is in progress by the use of a fluorescent reporter, thus, the reporter signal strength is directly proportional to the number of amplified copies [11]. These characteristics often make it the method of choice in routine diagnostics. Virus testing of imported propagation materials into the United States has been the most important measure used to prevent the introduction and spread of viruses [2], and real-time RT-PCR is one of the diagnostic tools employed by inspection agencies. There are two types of real-time RT-PCR systems. The first is based on a generic non-sequence-specific double-stranded DNA-binding dye such as SYBR Green, and the second is based on sequence-specific DNA hydrolysis probes [11]. In this study, we used TaqMan hydrolysis probes with a FAM dye label on the 5′ end and a minor groove binder (MGB) and nonfluorescent quencher on the 3′ end.

Here, currently available real-time RT-PCR assays for different *Prunus*-infecting viruses (Table 1) were evaluated, and in many cases updated or redesigned to accommodate additional sequence diversity that was not available at the time the assay was originally designed. In the case of viruses with no published real-time RT-PCR assay, a new assay was designed. Thus, 15 new or updated real-time RT-PCR assays were developed during this study. In most cases, these assays utilized multiple primers and probes for detecting all known virus variants. Comprehensive evaluation and compact design (i.e., use the minimum number of primers to cover the genetic diversity) of so many assays were made possible because of purpose built Python scripts. Subsequently, all assays were empirically validated using previously known infected plant material. Lastly, for additional validation, a *Prunus* germplasm collection, representing different accessions originating from 53 countries, was screened with the real-time RT-PCR assays.

## 2. Results

### 2.1. New or Updated Real-Time RT-PCR Assays That Accomodate Virus Genetic Diversity

Previously published real-time RT-PCR assays for targeted viruses (Table 1) were evaluated in silico to determine their capacity to detect the current virus isolates deposited in GenBank. In the case of ACLSV, CGRMV, cherry necrotic rusty mottle virus (CNRMV), cherry rasp leaf virus (CRLV), cherry virus A (CVA), LChV-1, LChV-2, plum bark necrosis stem pitting-associated virus (PBNSPaV), PDV, and PNRSV, our sequence analysis showed nucleotide mismatches between primers/probe sequences of corresponding assays and the alignment generated for each virus sequence. Mismatches observed during this analysis ranged from 1 to 10 nucleotides, highlighting the need to keep assays current with respect to known genetic diversity. In contrast, the CLRV assay did not display nucleotide mismatches, indicating that no modification was needed.

Additional primers or probes were added to the current ACLSV, CRLV, LChV-1, and PDV assays (Table 2; Figure S1) in order to cover all the known genetic diversity of the virus variants. Adjustments to these assays primarily involved one extra probe or up to two extra primers. Additionally, in the case of LChV-1, the degenerate oligonucleotide probe included in the original assay was replaced by two probes placed in a nearby conserved region.

Given the new sequence data available in GenBank, the in silico analysis revealed that the genomic regions targeted by the published CGRMV, CNRMV, CVA, LChV-2, PBNSPaV, and PNRSV assays were not as conserved as previously thought. As a consequence, new compact assays that amplified an alternative target were designed (Table 2; Figure S1).

Finally, real-time RT-PCR assays for cherry rusty mottle-associated virus (CRMaV), nectarine stem pitting-associated virus (NSPaV), nectarine virus M (NVM), and peach mosaic virus (PcMV) were not available. Compact real-time RT-PCR assays were developed for these viruses as described below (Table 2; Figure S1).

### 2.2. Detection of Targeted Viruses via High Throughput Sequencing

Select samples originating from the Foundation Plant Services (FPS) and the Clean Plant Center Northwest (CPCNW) collections of new *Prunus* introductions were analyzed for the viruses described in Table 1 using HTS. As a result of this inspection, multiple isolates were identified for all the viruses (Table S1), with the exception of NVM, PcMV, and CRMaV, which had only one isolate each. These *Prunus* samples were subsequently used to evaluate the updated/new assays (described below). If the HTS analysis determined that a sample was free of viruses or infected by not targeted viruses, the sample was used as a negative control.

### 2.3. Validation of Assay Design

Virus detection was validated by comparing real-time RT-PCR and HTS results for each virus-infected sample (Table S1). In all cases, real-time RT-PCR and HTS results agreed and Ct values were less than 28. No amplification was observed with healthy plant controls or plants infected by unrelated viruses, confirming the specificity of the assays. Since degenerate primers and probes with multiple sequence combinations (i.e., several possible bases in one or more positions) were not used to account for genetic diversity, the presence of all unique primers and TaqMan probes in the same reaction mixture was essential for the successful detection of all isolates in this study. The amplification efficiency varied among assays and ranged from 82% to 117% (Figure S2).

### 2.4. Screening of the Prunus Germplasm Collection

The real-time RT-PCR assays in Table 2 were used to evaluate the occurrence of viruses in a *Prunus* germplasm collection of diverse provenances at the National Clonal Germplasm Repository (NCGR). As a result of this survey, ACLSV, CGRMV, CNRMV, CVA, LChV-1, LChV-2, NSPaV, NVM, PcMV, PBNSPaV, PDV, or PNRSV were detected in 182 out of 333 trees or 54.6% of the tested accessions (Table 3; Table S2).

## 3. Discussion

This study exhaustively evaluated the genetic diversity represented by currently available *Prunus* fruit tree virus assays. We developed new and updated real-time RT-PCR assays to improve representation of current genetic diversity. These assays were designed with the dual goals of being compact, and at the same time, incorporating a complete picture of the known genetic diversity for high efficiency and sensitivity. New assays were designed to the most conserved region present in each virus species, which may involve the CP, RdRp, triple gene block 1, or the 3′ untranslated region. Among virus isolates, pairwise sequence similarity within assay regions varied between 88.5% to 100% (Table 4). In order to generate all these assays, custom scripts were utilized to accelerate and simplify assay design (e.g., sequence alignment and primer/probe design were completed in a single process). This also allowed us to use multiple variant matched primers and probes instead of single degenerate pairs. The smaller and more uniform primers in these assays are an attempt to ameliorate the lower efficiency degeneracy can lead to [12]; additionally, the use of degenerate primers may result in a major number of primers per reaction in comparison with our new assays.

*Prunus* samples from two collections, FPS and CPCNW, were used as virus sources to evaluate the updated or new assays. HTS analyses indicated that most of these samples (i.e., 69 out of 87) were infected with at least one of the 15 targeted viruses, revealing mixed infections in several samples (i.e., 20 samples). As a result of this evaluation, an agreement between real-time RT-PCR assay and HTS was obtained. To further validate the real-time RT-PCR assays, we collected and tested samples from the NCGR, which includes *Prunus* spp. accessions from a wide range of geographical regions. Although the actual virus diversity in the NCGR samples was not characterized by HTS, we detected 12 of the 15 viruses in 54.6% of the trees, suggesting that the PCR-based assays are robust. Thus, all the updated or new assays were tested against multiple isolates of each virus, except for CRMaV, which was identified in only one instance by both real-time RT-PCR and HTS.

During the initial validation of the real-time RT-PCR assays using samples previously analyzed by HTS, Ct values ranged from 12 to 28 and similar Ct values were obtained during the survey in the NCGR (Table S2). For CGRMV, CLRV, and PNRSV assays, there were a few cases where Ct values were >30. These samples were re-analyzed (i.e., extraction and testing were repeated) and confirmed to be negative. We hypothesize that these high Ct values were due to cross-contamination from strongly positive samples that were present in the initial processing. Consequently, any amplification after 30 cycles should be further investigated and verified.

In the United States, growers have adopted different methods for the control of viral diseases in fruit trees, including (i) the adoption of virus-tested propagation material and (ii) the eradication of infected trees [2]; all the viruses here investigated are part of the clean plant certification program. In that sense, new advances in real-time RT-PCR have significantly improved the detection of pathogens, allowing quick, sensitive, and precise identification compared to other historically used detection methods (e.g., end-point PCR, ELISA, and biological indexing). Moreover, real-time RT-PCR can be used to determine the number of virus copies present in a sample (i.e., virus quantification). In addition, it has the potential to be multiplexed with other assays, increasing testing efficiencies by identifying different viruses during the same reaction or by including an internal control. Thus, the development of highly sensitive real-time RT-PCR assays with broad-range detection capacity is needed for large scale testing of *Prunus* species that may be infected by the genetically diverse viruses included in this study. The assays developed here can help the clean stock programs and the fruit tree industry by facilitating early detection of virus-infected material. Likewise, Fotiou et al. [15] just published a new real-time RT-PCR for plum pox virus, which is considered as one of the most important pathogens in fruit trees and currently quarantined in the United States.

## 4. Materials and Methods

### 4.1. In Silico Analysis and Update of Available Real-Time RT-PCR Assays

The exhaustive evaluation, update, and design of 15 assays against the current version of GenBank was facilitated by purpose-built scripts implementing some of the procedures described below. For each of the viruses listed in Table 1, the most recently published real-time RT-PCR assay was first evaluated against all virus sequences deposited in GenBank. First, we used a BLAST [16] database search to identify and obtain all GenBank sequences overlapping the current assay region. To maximize sensitivity, a tBLASTn translated alignment exploiting codon redundancy was used. Highly divergent variants were further individually confirmed by separate BLAST analysis against GenBank to eliminate the possibility of misidentification. Once target sequences were collected and their species identification confirmed, all existing primers and probes were aligned to all target sequences from GenBank covering the assay region. This alignment was accomplished using a Perl script that used an end-gap-free nucleotide alignment to identify the best matching probe, forward and reverse primer sequences to each GenBank variant. In each case, the variant sequences corresponding to the matching oligos were collected and analyzed for divergence. Thus, all unique candidate sequence variants were inspected for total or partial divergence to an existing primer/probe sequence. The location and quantity of nucleotide differences and the frequency of the sequence in GenBank were also determined and assays were updated with extra primers or probes. One probe or one primer was added when more than two nucleotide mismatches were detected during the sequence comparison.

### 4.2. Development of New Compact Real-Time RT-PCR Assays

In a handful of cases where a real-time RT-PCR assay did not exist (i.e., CRMaV, NSPaV, NVM, and PcMV) or, on inspection of the current genetic diversity, the previous assay was impractical to extend and update (i.e., CGRMV, CNRMV, CVA, LChV-2, PBNSPaV, and PNRSV), a new assay was designed using an exhaustive approach that proposed a compact assay covering existing genetic diversity. To accomplish this objective, a Python script was used to minimize the size of the assay, with respect to the probe, the forward and reverse primer(s) sequences. First, an input multiple alignment **M** was determined. Conservation and depth of public sequence information across the virus genome was evaluated using a MUSCLE [17] multiple alignment of all virus sequences deposited in GenBank.

Let **M** be the m by n matrix containing the multiple sequence alignment considered for assay design. The matrix **M** contains n nucleotides or gap characters from each of m virus isolates. We define **M**(*i*,w) as the w adjacent columns of **M** starting at column *i*, and S(**M**,*i*,w) as the number of unique rows, aka sequences, in **M**(*i*,w). We wish to minimize S given the constraints of the design. For a proposed real-time RT-PCR probe width w_p_, an optimal location for the probe was determined by exhaustive search:

$$\underset{0\leq i\leq n}{\min}S\left(M,i,\;w\right)$$

Following optimal probe placement, we then considered the window of 100 bp to the left and right to obtain optimal forward and reverse primers. Let i_min_ be the optimal probe location, and w be the width of the proposed RT-PCR forward and reverse primers. Best candidate locations j_min_ and k_min_ for the forward and reverse primers were determined by sequential exhaustive searches:

$$\underset{\mathrm{imin}-100\;\leq \;j\;\leq \;\mathrm{imin}}{\min}S{\left(M,j,\;w\right)}_{\;}\;\;\;\;\underset{\mathrm{imin}+wp\;\leq \;k\;\leq \;\mathrm{imin}+150}{\min}S{\left(M,k,\;w\right)}_{\;}$$

Final determination of primer and probe sequences for a region included an additional more precise primer length adjustment step to the correct melting temperature (T_m_) according to the parameters for real-time RT-PCR with MGB probes employing the Primer Express software (ThermoFisher Scientific, Foster City, CA, USA). Primer–primer and primer–probe interactions were also evaluated using the same software. Finally, primers and probes were ranked by their frequency in the database. Considering the list of primers/probes in order of decreasing frequency, all primers and probes contributing two or more additional nucleotides, or one nucleotide within two bases of the 3′ end, were included in the final assay design. In general, the nucleotide sequence identity among virus isolates included in the assay regions varied between 88.5% to 100% (Table 4).

### 4.3. Virus Screening via High Throughput Sequencing

Plant material reported or suspected to be infected by the studied viruses was obtained from FPS (University of California-Davis) and the CPCNW (Washington State University); both *Prunus* collections include foreign and domestic introductions. In total, 87 *Prunus* samples (Table 5) were obtained and included in the virus screening via HTS. Briefly, total nucleic acid (TNA) extracts from *Prunus* samples were prepared following the methodology described by Al Rwahnih et al. [18]. Later, TNA aliquots were subjected to ribosomal RNA (rRNA) depletion and complementary DNA library construction using the TruSeq Stranded Total RNA with Ribo-Zero Plant kit (Illumina, San Diego, CA, USA). HTS analysis for known viruses was accomplished as described in [19], but the de novo assembly was completed by SPAdes v3.11 [20].

### 4.4. Initial Validation of Assay Design and Efficiency

All the assays described in Table 2 were challenged against the set of 87 *Prunus* samples previously analyzed by HTS; such set included different viruses and multiple isolates of each virus, except for NVM, PcMV, and CRMaV with one isolate only. In addition, samples free of targeted viruses were considered in the analysis as negative controls.

Real-time RT-PCR reactions were completed in the QuantStudio 6 real-time PCR system using the TaqMan Fast Virus 1-Step Master Mix (ThermoFisher Scientific, Foster City, CA) and following the recommended protocol. Each reaction (10 µL final volume) included 2 µL of TNA and final primer and probe concentrations of 900 and 250 ῃM, respectively. The thermocycler conditions were as follows: 50 °C for 5 min, 95 °C for 20 s, followed by 40 cycles of 95 °C for 3 s and 60 °C for 30 s. Additionally, the assays were multiplexed with a previously published 18S rRNA assay [21] to verify the presence of high-quality RNA during the reaction.

The efficiency of each real-time RT-PCR assay was determined using serial dilutions (1:1 to 1:1,000,000) of TNA extracts in water and run in triplicate. Standard curves were calculated using the QuantStudio 6 real-time PCR software.

### 4.5. Survey in the NCGR

The NCGR, a United States Department of Agriculture genetic resource, is located near Winters, California, and contains approximately 4000 *Prunus* trees representing different accessions (https://npgsweb.ars-grin.gov). Trees in this collection originate globally and contain almonds, apricots, cherries, peaches, plums, and nectarines. Using a random methodology but taking in consideration different types of *Prunus* material and countries of origin, 333 accessions (Table 6) were sampled and later tested via real-time RT-PCR.

## Figures and Tables

**Table 1:** *Prunus*-infecting viruses included in this study and currently available real-time RT-PCR assays.

Virus	Acronym	Assay Citation
Apple chlorotic leafspot virus	ACLSV	Osman et al. 2016 [10]
Cherry green ring mottle virus	CGRMV	Osman et al. 2016 [10]
Cherry leaf roll virus	CLRV	Osman et al. 2014 [9]
Cherry necrotic rusty mottle virus	CNRMV	Osman et al. 2016 [10]
Cherry rasp leaf virus	CRLV	Osman et al. 2016 [10]
Cherry rusty mottle-associated virus	CRMaV	NA
Cherry virus A	CVA	Osman et al. 2016 [10]
Little cherry virus 1	LChV-1	Katsiani et al. 2017 [12]
Little cherry virus 2	LChV-2	Jelkmann et al. 2006 [13]
Nectarine stem pitting-associated virus	NSPaV	NA
Nectarine virus M	NVM	NA
Peach mosaic virus	PcMV	NA
Plum bark necrosis stem pitting-associated virus	PBNSPaV	Lin et al. 2013 [14]
Prune dwarf virus	PDV	Osman et al. 2014 [9]
Prunus necrotic ringspot virus	PNRSV	Osman et al. 2014 [9]

Not available assay (NA).

**Table 2:** Updated or newly designed assays for detection of *Prunus*-infecting viruses.

Virus ^1^	Oligo Name ^2^	Sequence (5′ to 3′) ^3^	5′ Reporter	Probe Type	Target Region ^4^	Reference
ACLSV	ACLSV-F1	**GCAGACCCCTTCATGGAAAG**			CP	Osman et al., 2016 [10]
	ACLSV-R1	**TTCGGGTCCGAAGATGTAGTC**				
	ACLSV-R2	**TTCGGGTCCGAAGAGGTAGTC**				
	ACLSV-R3	**TGTTCGGATCCGAAGATGTAGTC**				
	ACLSV-R4	**TGTTTGGGTCCGAAGATGTAGTC**				
	ACLSV-R5	GATGTTCAAATCCGAAGAGGTAGTC				
	ACLSV-P1	**CCATCTTCGCGAACAT**	FAM	MGB		
	ACLSV-P2	**CCATCTTCGCGAATAT**	FAM	MGB		
CGRMV	CGRMV-F1	GCCTGGTTGCGGGAAAT			TGB1	This study
	CGRMV-F2	GCCTGGCTGCGGGAA				
	CGRMV-R1	GGGCGTGAAAGTCCTCAAGA				
	CGRMV-P1	CTCTTGTCAGGAAGTTT	FAM	MGB		
CLRV	CLRV-F1	**TGGCGACCGTGTAACGG**			3′ UTR	Osman et al., 2014 [9]
	CLRV-R1	**TACTACTAAGACCGGTCGCATGG**				
	CLRV-R2	**TACTACTAAGACCGGTCGCATGAA**				
	CLRV-P1	**GTTAAGGTGACACTGGTGG**	FAM	MGB		
	CLRV-P2	**TTACGGTGACACTGGTGG**	FAM	MGB		
CNRMV	CNRMV-F1	AATCCCACCTCAAGTCCTAGCAG			CP	This study
	CNRMV-R1	GTGCTCAACCCAATCGGC				
	CNRMV-P1	GACCCTACAACTCTCAACAT	FAM	MGB		
CRLV	CRLV-F1	**TGCGTTCCAAAGGGACAAA**			RdRp	Osman et al., 2016 [10]
	CRLV-R1	**TCCTGGGCGTAATCCCATC**				
	CRLV-R2	AAACATTCCTTGGTGTTATTCCATC				
	CRLV-P1	**TGGTTTAATGGTGATTATTC**	FAM	MGB		
	CRLV-P2	TGCTTTAATGGTGTTTATTC	FAM	MGB		
CRMaV	CRMaV-F1	TAATTGCATCTTTGATGTTGTCTGG			CP	This study
	CRMaV-F2	TTTAATTGCATCTTTGATATTGTCTGG				
	CRMaV-R1	TGCGTAGAGAGCAGTAGCTCCTAAC				
	CRMaV-R2	TGCGTAAAGAGCAGTAGCTCCTAAC				
	CRMaV-P1	TGTTATCATAACAGCTCCAG	FAM	MGB		
CVA	CVA-F1	CCGAGACCGGTGATAGAGAATC			CP	This study
	CVA-F2	CCGAGACCAGTGATAGAGAATCAG				
	CVA-F3	CCGAGACCAGTGATAGAGAATCAA				
	CVA-R1	GCACCAACTACACCCCATGC				
	CVA-R2	GCACCAACCACACCCCA				
	CVA-P1	ACTGCACATCTCCCAGC	FAM	MGB		
	CVA-P2	ACTGCGCATCTCCCAG	FAM	MGB		
LChV-1	LChV1-F1	**CCAATGCACAAAGCACATATGA**			CP	Katsiani et al., 2017 [12]
	LChV1-F2	**CCAATGCATAAAGCTCATATGACAT**				
	LChV1-F3	CCGATGCACAAAGCATCAAT				
	LChV1-F4	CGATGCATAAAGCTCATATGACGT				
	LChV1-R1	**CTTGCGAAACATGAAGAGCTCC**				
	LChV1-P1	GATACTGATACGTCTAGCTCG	FAM	MGB		
	LChV1-P2	GATACTGATACGACTAGCTCG	FAM	MGB		
LChV-2	LChV2-F1	TTTGACCCGAATACCTTCGTG			RdRp	This study
	LChV2-F2	AGTTCGACCCGAATACTTTTGTG				
	LChV2-R1	TACAAAAGTATGGAGTTGCAACAGG				
	LChV2-P1	TTCTGGAGATGATTCATT	FAM	MGB		
	LChV2-P2	TTCAGGAGACGATTCTT	FAM	MGB		
NSPaV	NSPaV-F1	AGCGAATGGAGCAAAATCTGA			CP	This study
	NSPaV-F2	AAAGCAAATGGAGCAAAATCTGAT				
	NSPaV-R1	CAATGAGTGTGCAGGGTGATG				
	NSPaV-R2	CAGTGAGTGTGCAGGGTGATG				
	NSPaV-P1	TCGCTGGGCAATTT	FAM	MGB		
NVM	NVM-F1	TGATTCCCTCCTCGACTACGA			RdRp Polyprotein	This study
	NVM-R1	AGGCTTGATGGCGTTCCA				
	NVM-R2	GAGGCTTAATGGCGTTCCAC				
	NVM-P1	CCCAAGGTCCGACCC	FAM	MGB		
PcMV	PcMV-F1	ACGAGGATGGCTCTGATGATG			RdRp	This study
	PcMV-R1	ACAAACTCACTCCAATGGATCATC				
	PcMV-R2	GCAAACTCACTCCAGTGGATCAT				
	PcMV-P1	TTTCTGGAGTGAAAAGC	FAM	MGB		
PBNSPaV	PBNSPaV-F1	GGTGTAAGTCTTGAGCCTCTTTTCTG			3′ UTR	This study
	PBNSPaV-R1	ACCACCCGAGACAGGTGATTT				
	PBNSPaV-P1	CTGTTCTCCGAACAGATAA	FAM	MGB		
PDV	PDV-F1	**TGATACCAAGGTATACGGAATTG**			CP	Osman et al., 2014 [9]
	PDV-F2	**TGATACCAAGGTATACGGAATCG**				
	PDV-F3	**TGATACCAAGGTATACGGGATTGC**				
	PDV-F4	**TGATACCAAGGTGTACGGAATAGTTT**				
	PDV-R1	**TGAACTTCCTACGTTGTAGGGGAT**				
	PDV-R2	AAACTTCCTCCTAGAGAGGGGATT				
	PDV-P1	**TCTACGGACTCATTAAAGGT**	FAM	MGB		
	PDV-P2	TGTTTACGGACTCATTAAA	FAM	MGB		
PNRSV	PNRSV-F1	ACCGAGAGGTGACAACGACAG			CP	This study
	PNRSV-F2	CACCGAGAGGTGACGACGA				
	PNRSV-F3	ACCGAGAGGTGATGACGACAG				
	PNRSV-F4	CACCGTGAGGTGACGACTACTG				
	PNRSV-R1	CCTTCAAGAACCCCTTCCTAGAC				
	PNRSV-R2	CCTTCAGAAAACCCTTCCTAGACA				
	PNRSV-P1	CCGAATGAACTCTATGAGTT	FAM	MGB		
	PNRSV-P2	CCGAATGAACTCAAGGAG	FAM	MGB		

^1^ Virus name and corresponding acronym in Table 1. ^2^ Forward primer (F), reverse primer (R), and probe (P). ^3^ Sequences in bold represent the primers and probes included in previously published assays. ^4^ Coat protein (CP), RNA-dependent RNA polymerase (RdRp), triple gene block 1 (TGB1), untranslated region (UTR).

**Table 3:** Viruses identified during the survey in the National Clonal Germplasm Repository.

Virus	Number of Infected Trees
ACLSV	19 (5.7%)
CGRMV	20 (6%)
CLRV	0 (0%)
CNRMV	4 (1.2%)
CRLV	0 (0%)
CRMaV	0 (0%)
CVA	39 (11.7%)
LChV-1	10 (3%)
LChV-2	3 (0.9%)
NSPaV	4 (1.2%)
NVM	10 (3%)
PcMV	2 (0.6%)
PBNSPaV	33 (9.9%)
PDV	29 (8.7%)
PNRSV	127 (38.1%)

**Table 4:** Comparison between genome regions amplified by each real-time RT-PCR assay and GenBank accessions included in assay design.

Assay	Amplicon Size	Identity of Target Region	Number of Accessions Included in Design
ACLSV	218 bp	88.5%	247
CGRMV	62 bp	96.5%	35
CLRV	83 bp	97.3%	46
CNRMV	120 bp	97.3%	80
CRLV	72 bp	93%	6
CRMaV	139 bp	97%	23
CVA	107 bp	96.6%	67
LChV-1	115 bp	90.3%	13
LChV-2	147 bp	94.1%	6
NSPaV	62 bp	98.9%	6
NVM	59 bp	98.9%	4
PcMV	152 bp	95.4%	7
PBNSPaV	71 bp	100%	10
PDV	127 bp	95.6%	122
PNRSV	216 bp	95.6%	230

**Table 5:** *Prunus* samples analyzed by high throughput sequencing during this study.

Prunus Tree	Number of Samples
Almond	3
Apricot	2
Cherry	45
Nectarine	10
Peach	25
Plum	2

**Table 6:** *Prunus* accessions included in the survey and corresponding country of origin.

Country of Origin	Number of Accessions
Afghanistan	2
Albania	3
Armenia	4
Australia	3
Azerbaijan	3
Belgium	1
Bosnia and Herzegovina	1
Brazil	5
Bulgaria	4
Canada	7
China	17
Czech Republic	2
Former Serbia and Montenegro	7
France	7
Georgia	5
Germany	4
Greece	2
Guatemala	1
Hungary	7
India	8
Iran	2
Israel	1
Italy	11
Japan	10
Kazakhstan	5
South Korea	3
Kyrgyzstan	1
Latvia	1
Lebanon	1
Malta	1
Mexico	5
Morocco	3
Nepal	4
Netherlands	3
New Zealand	5
Pakistan	23
Poland	6
Romania	10
Russian Federation	8
Serbia	1
South Africa	7
Spain	3
Sweden	2
Switzerland	2
Syria	1
Taiwan	5
Thailand	4
Turkey	7
Turkmenistan	4
Ukraine	5
United Kingdom	7
United States	75
Uzbekistan	7
Unknown	7

## Supplementary Materials

The following material are available online at https://www.mdpi.com/2223-7747/9/2/273/s1. Figure S1: Alignments of genomic regions present in different Prunus-infecting viruses and amplified by the real-time RT-PCR assays. Figure S2: Amplification plot and standard curve generated from new/updated real-time RT-PCR assays. Table S1: Prunus samples analyzed by HTS and included in the initial validation of assays. Table S2: Information of samples collected at the NCGR and tested positive for viruses during the survey.
