# Supplementary material for: Comprehensive Real-Time RT-PCR Assays for the Detection of Fifteen Viruses Infecting Prunus spp
Source: Plants (Basel). 2020 Feb 19;9(2):273. doi: 10.3390/plants9020273 (PMC7076543; doi:10.3390/plants9020273)
Supplement: Supplementary file 1 [file plants-09-00273-s001.zip › Figure S1.pdf]

Figure S1. Alignments of genomic regions present in different *Prunus*-infecting viruses and amplified by the real-time RT-PCR assays. Multiple virus isolates available in GenBank were employed for assay design; accession numbers listed on the left side of each alignment, in the case of ACLSV, CNRMV, CVA, PDV and PNRSV a subset of analyzed accessions is displayed only. Primers and probes included in the assays are highlighted in red color and located at the bottom of each alignment. Alignments generated with MUSCLE and Geneious programs.

CGRMV

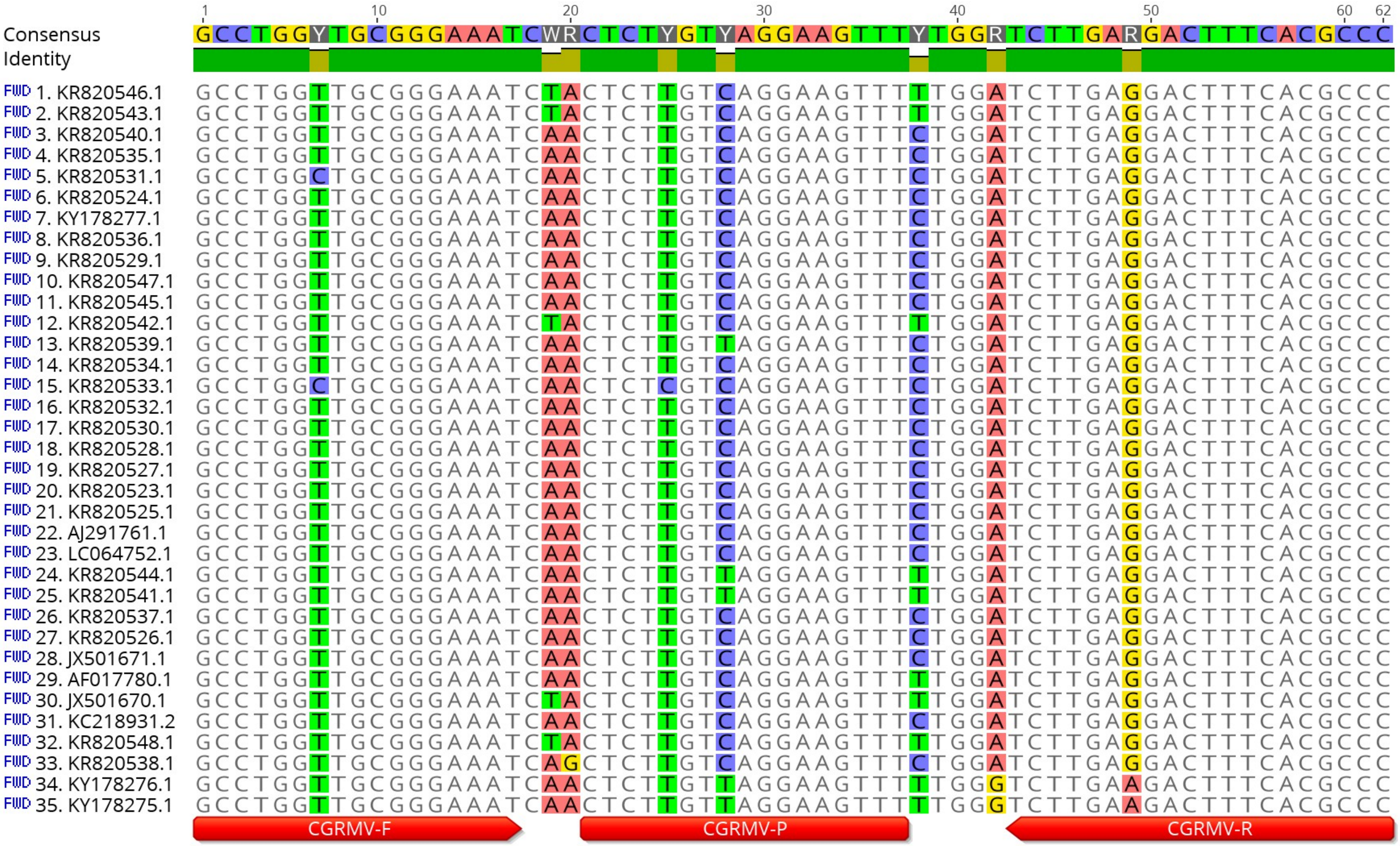

ACLSV

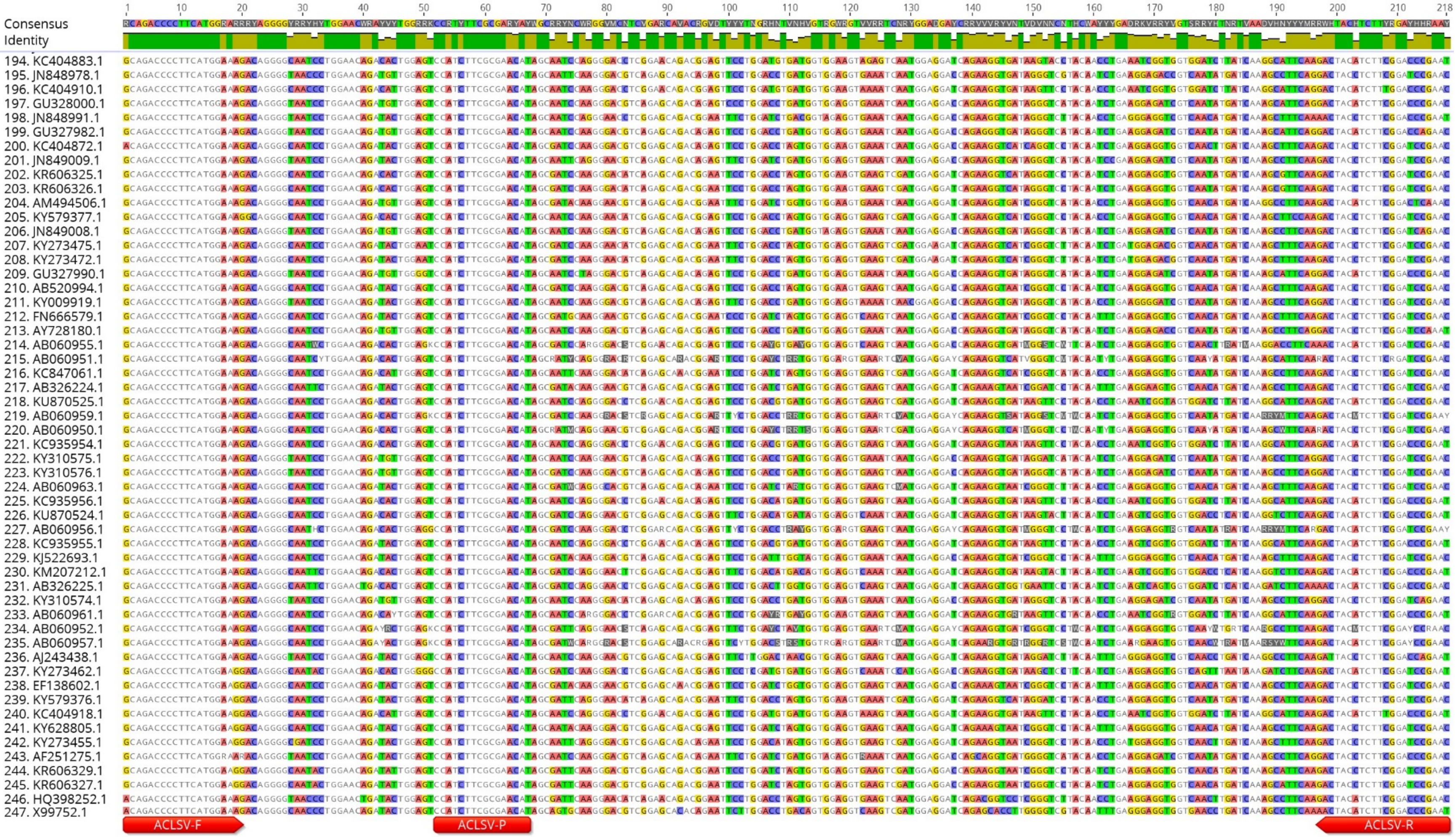

Consensus
Identity
194. KC404883.1
195. JN848978.1
196. KC404910.1
197. GU328000.1
198. JN848991.1
199. GU327982.1
200. KC404872.1
201. JN849009.1
202. KR606325.1
203. KR606326.1
204. AM494506.1
205. KY579377.1
206. JN849008.1
207. KY273475.1
208. KY273472.1
209. GU327990.1
210. AB520994.1
211. KY009919.1
212. FN666579.1
213. AY728180.1
214. AB060955.1
215. AB060951.1
216. KC847061.1
217. AB326224.1
218. KU870525.1
219. AB060959.1
220. AB060950.1
221. KC935954.1
222. KY310575.1
223. KY310576.1
224. AB060963.1
225. KC935956.1
226. KU870524.1
227. AB060956.1
228. KC935955.1
229. KJ522693.1
230. KM207212.1
231. AB326225.1
232. KY310574.1
233. AB060961.1
234. AB060952.1
235. AB060957.1
236. AJ243438.1
237. KY273462.1
238. EF138602.1
239. KY579376.1
240. KC404918.1
241. KY628805.1
242. KY273455.1
243. AF251275.1
244. KR606329.1
245. KR606327.1
246. HQ398252.1
247. X99752.1
ACLSV-F
ACLSV-P
ACLSV-R

CLRV

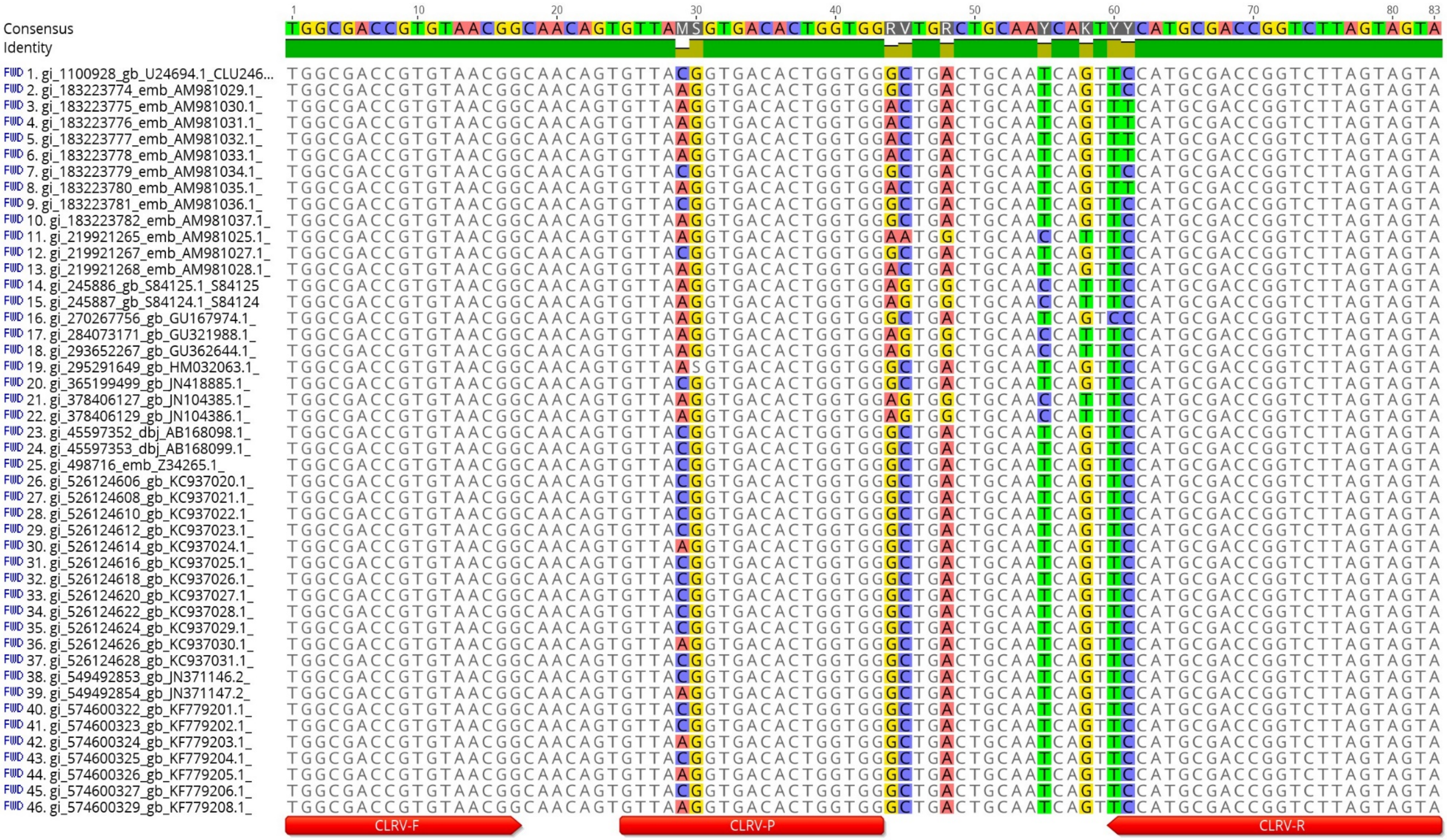
Consensus
TGGCGACCGTGTAACGGCAACAGTGTTAMSGTGACACTGGTGGRVTGRCTGCAAYCAKTYYCATGCGACCGGTCTTAGTAGTA
Identity
FWD 1. gi_1100928_gb_U24694.1_CLU246...
FWD 2. gi_183223774_emb_AM981029.1_
FWD 3. gi_183223775_emb_AM981030.1_
FWD 4. gi_183223776_emb_AM981031.1_
FWD 5. gi_183223777_emb_AM981032.1_
FWD 6. gi_183223778_emb_AM981033.1_
FWD 7. gi_183223779_emb_AM981034.1_
FWD 8. gi_183223780_emb_AM981035.1_
FWD 9. gi_183223781_emb_AM981036.1_
FWD 10. gi_183223782_emb_AM981037.1_
FWD 11. gi_219921265_emb_AM981025.1_
FWD 12. gi_219921267_emb_AM981027.1_
FWD 13. gi_219921268_emb_AM981028.1_
FWD 14. gi_245886_gb_S84125.1_S84125
FWD 15. gi_245887_gb_S84124.1_S84124
FWD 16. gi_270267756_gb_GU167974.1_
FWD 17. gi_284073171_gb_GU321988.1_
FWD 18. gi_293652267_gb_GU362644.1_
FWD 19. gi_295291649_gb_HM032063.1_
FWD 20. gi_365199499_gb_JN418885.1_
FWD 21. gi_378406127_gb_JN104385.1_
FWD 22. gi_378406129_gb_JN104386.1_
FWD 23. gi_45597352_dbj_AB168098.1_
FWD 24. gi_45597353_dbj_AB168099.1_
FWD 25. gi_498716_emb_Z34265.1_
FWD 26. gi_526124606_gb_KC937020.1_
FWD 27. gi_526124608_gb_KC937021.1_
FWD 28. gi_526124610_gb_KC937022.1_
FWD 29. gi_526124612_gb_KC937023.1_
FWD 30. gi_526124614_gb_KC937024.1_
FWD 31. gi_526124616_gb_KC937025.1_
FWD 32. gi_526124618_gb_KC937026.1_
FWD 33. gi_526124620_gb_KC937027.1_
FWD 34. gi_526124622_gb_KC937028.1_
FWD 35. gi_526124624_gb_KC937029.1_
FWD 36. gi_526124626_gb_KC937030.1_
FWD 37. gi_526124628_gb_KC937031.1_
FWD 38. gi_549492853_gb_JN371146.2_
FWD 39. gi_549492854_gb_JN371147.2_
FWD 40. gi_574600322_gb_KF779201.1_
FWD 41. gi_574600323_gb_KF779202.1_
FWD 42. gi_574600324_gb_KF779203.1_
FWD 43. gi_574600325_gb_KF779204.1_
FWD 44. gi_574600326_gb_KF779205.1_
FWD 45. gi_574600327_gb_KF779206.1_
FWD 46. gi_574600329_gb_KF779208.1_
CLRV-F
CLRV-P
CLRV-R

CNRMV

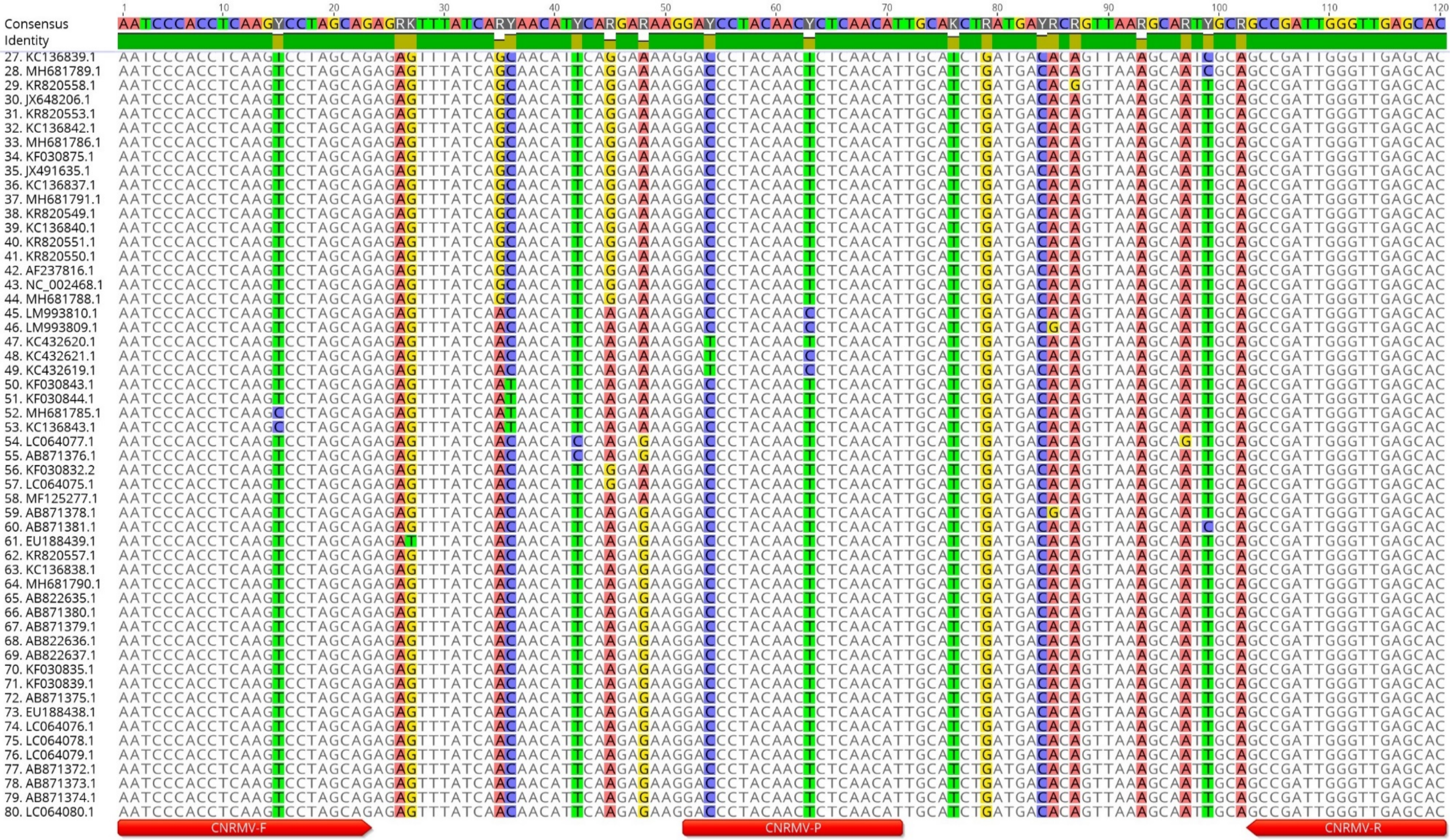
Consensus
Identity
1
10
20
30
40
50
60
70
80
90
100
110
120
27. KC136839.1
28. MH681789.1
29. KR820558.1
30. JX648206.1
31. KR820553.1
32. KC136842.1
33. MH681786.1
34. KF030875.1
35. JX491635.1
36. KC136837.1
37. MH681791.1
38. KR820549.1
39. KC136840.1
40. KR820551.1
41. KR820550.1
42. AF237816.1
43. NC_002468.1
44. MH681788.1
45. LM993810.1
46. LM993809.1
47. KC432620.1
48. KC432621.1
49. KC432619.1
50. KF030843.1
51. KF030844.1
52. MH681785.1
53. KC136843.1
54. LC064077.1
55. AB871376.1
56. KF030832.2
57. LC064075.1
58. MF125277.1
59. AB871378.1
60. AB871381.1
61. EU188439.1
62. KR820557.1
63. KC136838.1
64. MH681790.1
65. AB822635.1
66. AB871380.1
67. AB871379.1
68. AB822636.1
69. AB822637.1
70. KF030835.1
71. KF030839.1
72. AB871375.1
73. EU188438.1
74. LC064076.1
75. LC064078.1
76. LC064079.1
77. AB871372.1
78. AB871373.1
79. AB871374.1
80. LC064080.1
CNRMV-F
CNRMV-P
CNRMV-R

CRLV

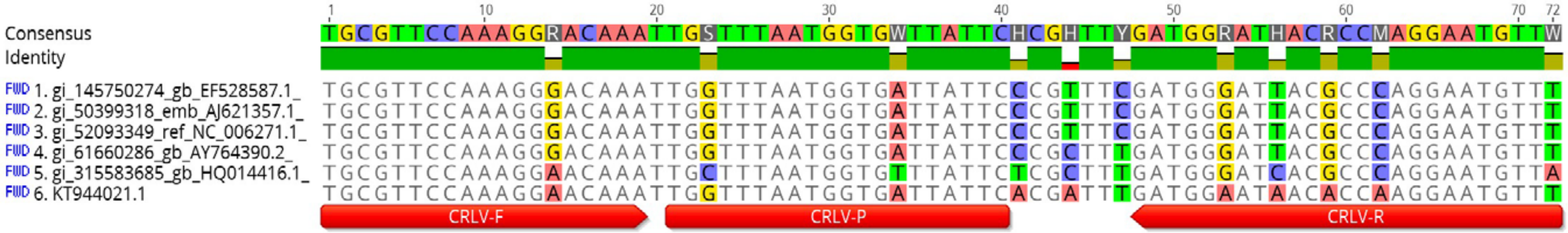

Consensus
Identity
FWD 1. gi_145750274_gb_EF528587.1_
FWD 2. gi_50399318_emb_AJ621357.1_
FWD 3. gi_52093349_ref_NC_006271.1_
FWD 4. gi_61660286_gb_AY764390.2_
FWD 5. gi_315583685_gb_HQ014416.1_
FWD 6. KT944021.1
CRLV-F
CRLV-P
CRLV-R

CRMaV

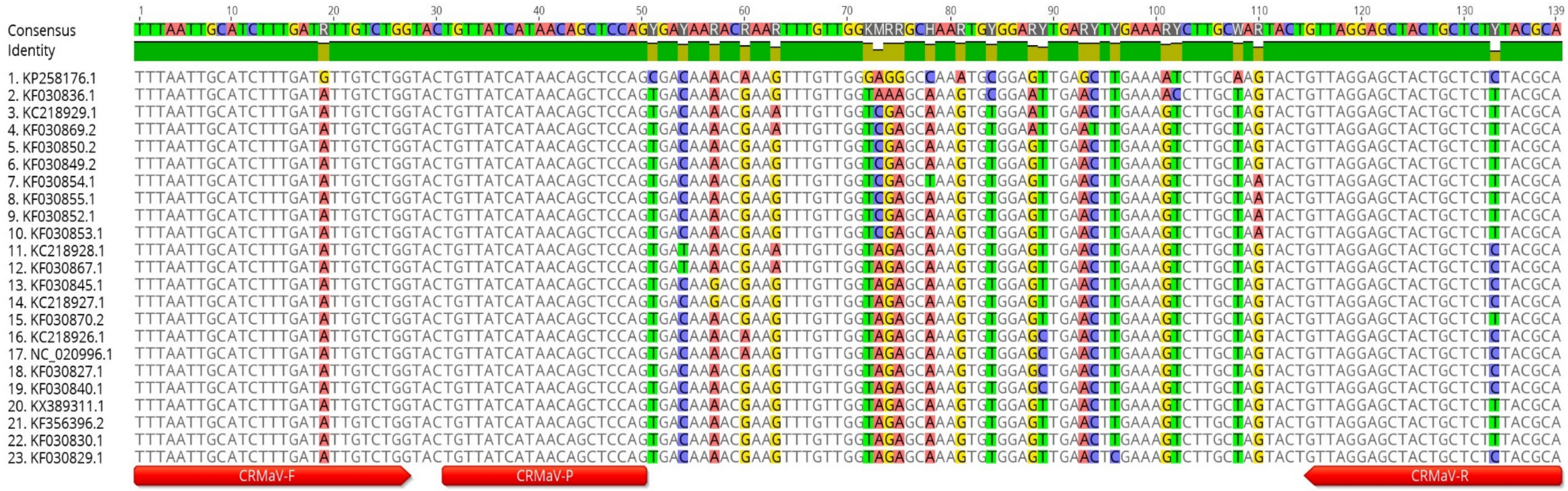
Consensus
Identity
1. KP258176.1
2. KF030836.1
3. KC218929.1
4. KF030869.2
5. KF030850.2
6. KF030849.2
7. KF030854.1
8. KF030855.1
9. KF030852.1
10. KF030853.1
11. KC218928.1
12. KF030867.1
13. KF030845.1
14. KC218927.1
15. KF030870.2
16. KC218926.1
17. NC_020996.1
18. KF030827.1
19. KF030840.1
20. KX389311.1
21. KF356396.2
22. KF030830.1
23. KF030829.1
CRMaV-F
CRMaV-P
CRMaV-R

CVA

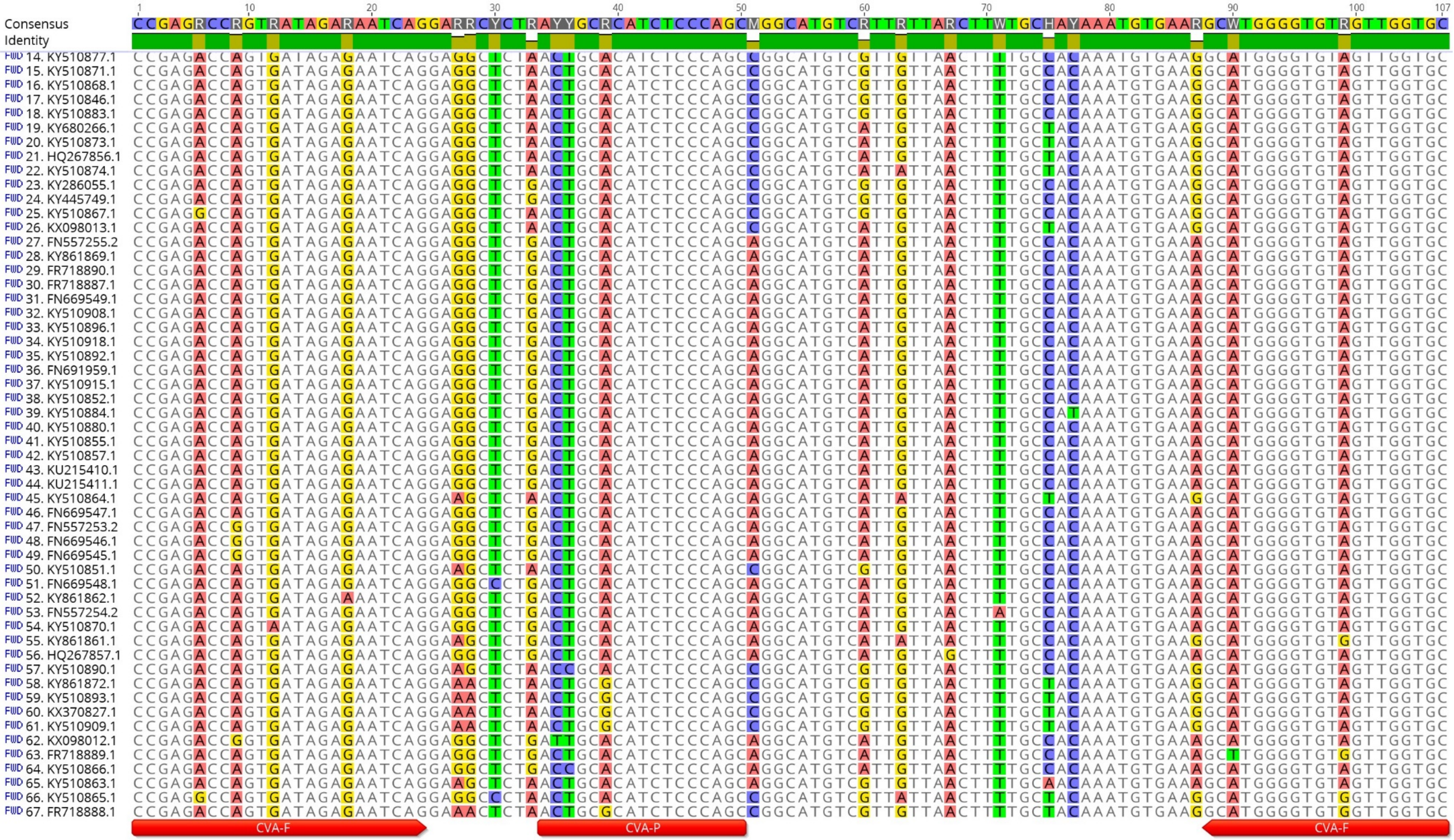

Consensus
Identity
1
10
20
30
40
50
60
70
80
90
100
107
FMD 14. KY510877.1
FMD 15. KY510871.1
FMD 16. KY510868.1
FMD 17. KY510846.1
FMD 18. KY510883.1
FMD 19. KY680266.1
FMD 20. KY510873.1
FMD 21. HQ267856.1
FMD 22. KY510874.1
FMD 23. KY286055.1
FMD 24. KY445749.1
FMD 25. KY510867.1
FMD 26. KX098013.1
FMD 27. FN557255.2
FMD 28. KY861869.1
FMD 29. FR718890.1
FMD 30. FR718887.1
FMD 31. FN669549.1
FMD 32. KY510908.1
FMD 33. KY510896.1
FMD 34. KY510918.1
FMD 35. KY510892.1
FMD 36. FN691959.1
FMD 37. KY510915.1
FMD 38. KY510852.1
FMD 39. KY510884.1
FMD 40. KY510880.1
FMD 41. KY510855.1
FMD 42. KY510857.1
FMD 43. KU215410.1
FMD 44. KU215411.1
FMD 45. KY510864.1
FMD 46. FN669547.1
FMD 47. FN557253.2
FMD 48. FN669546.1
FMD 49. FN669545.1
FMD 50. KY510851.1
FMD 51. FN669548.1
FMD 52. KY861862.1
FMD 53. FN557254.2
FMD 54. KY510870.1
FMD 55. KY861861.1
FMD 56. HQ267857.1
FMD 57. KY510890.1
FMD 58. KY861872.1
FMD 59. KY510893.1
FMD 60. KX370827.1
FMD 61. KY510909.1
FMD 62. KX098012.1
FMD 63. FR718889.1
FMD 64. KY510866.1
FMD 65. KY510863.1
FMD 66. KY510865.1
FMD 67. FR718888.1
CVA-F
CVA-P
CVA-F

LChV-1

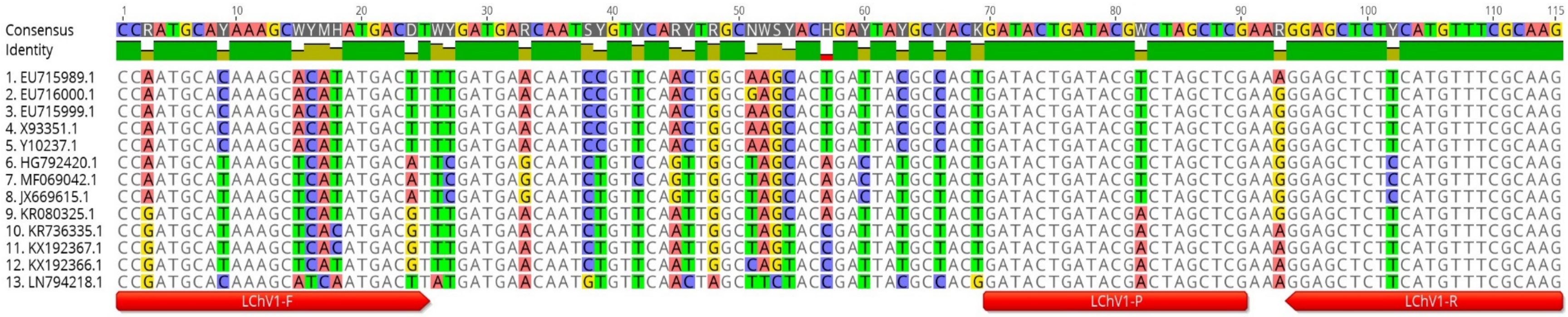
Consensus
Identity
1. EU715989.1
2. EU716000.1
3. EU715999.1
4. X93351.1
5. Y10237.1
6. HG792420.1
7. MF069042.1
8. JX669615.1
9. KR080325.1
10. KR736335.1
11. KX192367.1
12. KX192366.1
13. LN794218.1
LChV1-F
LChV1-P
LChV1-R

LChV-2

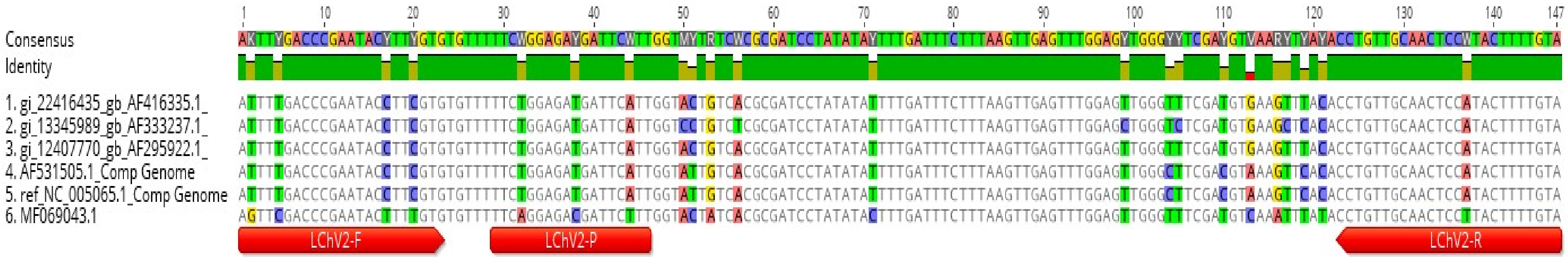
Consensus
Identity
1. gi_22416435_gb_AF416335.1_
2. gi_13345989_gb_AF333237.1_
3. gi_12407770_gb_AF295922.1_
4. AF531505.1_Comp Genome
5. ref_NC_005065.1_Comp Genome
6. MF069043.1
LChV2-F
LChV2-P
LChV2-R

NSPaV

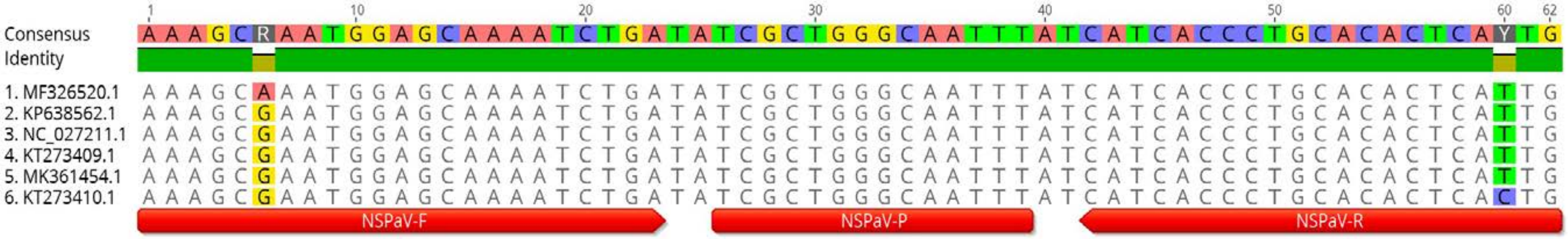
Consensus
Identity
1. MF326520.1
2. KP638562.1
3. NC_027211.1
4. KT273409.1
5. MK361454.1
6. KT273410.1
NSPaV-F
NSPaV-P
NSPaV-R

NVM

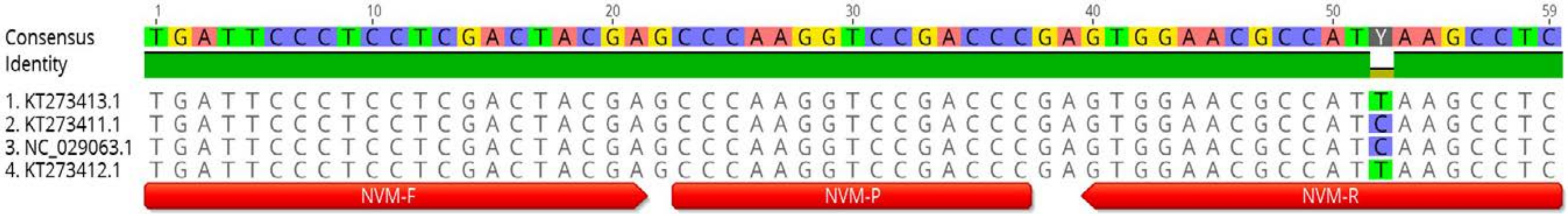

1
10
20
30
40
50
59
Consensus
Identity
TGATTCCCTCCTCGACTACGAGCCCAAGGTCCGACCCGAGTGGAACGCCATYAAGCCTC
1. KT273413.1 TGATTCCCTCCTCGACTACGAGCCCAAGGTCCGACCCGAGTGGAACGCCATTAAGCCTC
2. KT273411.1 TGATTCCCTCCTCGACTACGAGCCCAAGGTCCGACCCGAGTGGAACGCCATCAAGCCTC
3. NC_029063.1 TGATTCCCTCCTCGACTACGAGCCCAAGGTCCGACCCGAGTGGAACGCCATCAAGCCTC
4. KT273412.1 TGATTCCCTCCTCGACTACGAGCCCAAGGTCCGACCCGAGTGGAACGCCATTAAGCCTC
NVM-F
NVM-P
NVM-R

PcMV

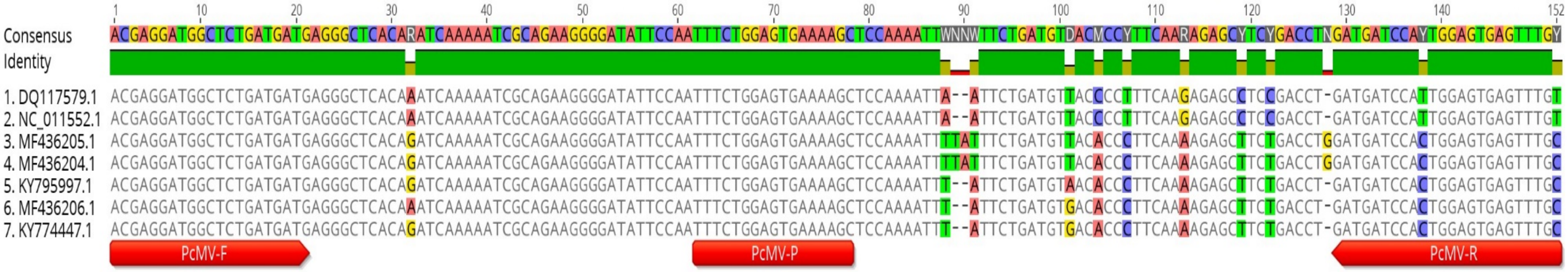
Consensus
Identity
1. DQ117579.1
2. NC_011552.1
3. MF436205.1
4. MF436204.1
5. KY795997.1
6. MF436206.1
7. KY774447.1
PcMV-F
PcMV-P
PcMV-R

PBNSPaV

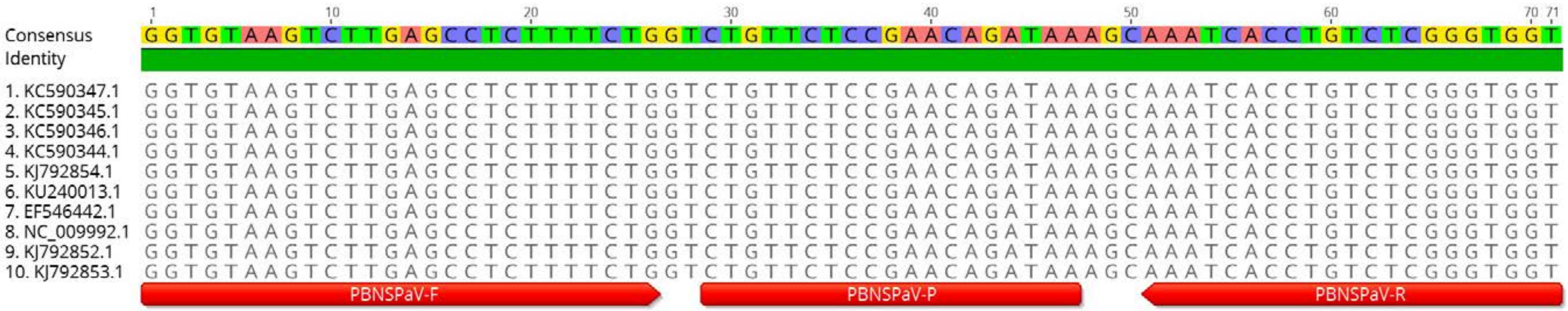

1 10 20 30 40 50 60 70 71
Consensus GGTGTAAGTCTTGAGCCTCTTTTCTGGTCTGTTCTCCGAACAGATAAAGCAAATCACCTGTCTCGGGTGGT
Identity
1. KC590347.1 GGTGTAAGTCTTGAGCCTCTTTTCTGGTCTGTTCTCCGAACAGATAAAGCAAATCACCTGTCTCGGGTGGT
2. KC590345.1 GGTGTAAGTCTTGAGCCTCTTTTCTGGTCTGTTCTCCGAACAGATAAAGCAAATCACCTGTCTCGGGTGGT
3. KC590346.1 GGTGTAAGTCTTGAGCCTCTTTTCTGGTCTGTTCTCCGAACAGATAAAGCAAATCACCTGTCTCGGGTGGT
4. KC590344.1 GGTGTAAGTCTTGAGCCTCTTTTCTGGTCTGTTCTCCGAACAGATAAAGCAAATCACCTGTCTCGGGTGGT
5. KJ792854.1 GGTGTAAGTCTTGAGCCTCTTTTCTGGTCTGTTCTCCGAACAGATAAAGCAAATCACCTGTCTCGGGTGGT
6. KU240013.1 GGTGTAAGTCTTGAGCCTCTTTTCTGGTCTGTTCTCCGAACAGATAAAGCAAATCACCTGTCTCGGGTGGT
7. EF546442.1 GGTGTAAGTCTTGAGCCTCTTTTCTGGTCTGTTCTCCGAACAGATAAAGCAAATCACCTGTCTCGGGTGGT
8. NC_009992.1 GGTGTAAGTCTTGAGCCTCTTTTCTGGTCTGTTCTCCGAACAGATAAAGCAAATCACCTGTCTCGGGTGGT
9. KJ792852.1 GGTGTAAGTCTTGAGCCTCTTTTCTGGTCTGTTCTCCGAACAGATAAAGCAAATCACCTGTCTCGGGTGGT
10. KJ792853.1 GGTGTAAGTCTTGAGCCTCTTTTCTGGTCTGTTCTCCGAACAGATAAAGCAAATCACCTGTCTCGGGTGGT
PBNSPaV-F
PBNSPaV-P
PBNSPaV-R

PDV

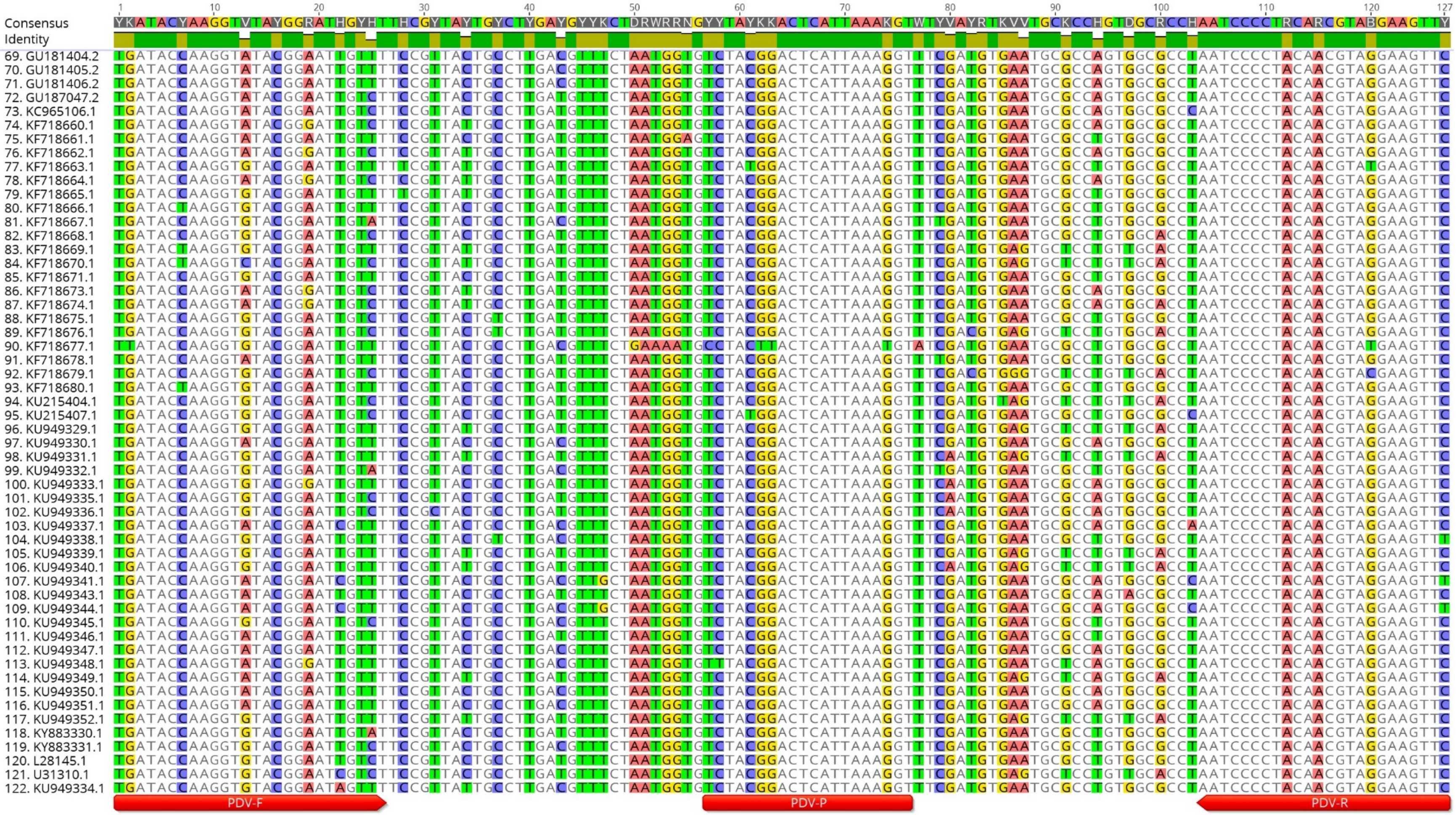

1
10
20
30
40
50
60
70
80
90
100
110
120
127
Consensus
Identity
69. GU181404.2
70. GU181405.2
71. GU181406.2
72. GU187047.2
73. KC965106.1
74. KF718660.1
75. KF718661.1
76. KF718662.1
77. KF718663.1
78. KF718664.1
79. KF718665.1
80. KF718666.1
81. KF718667.1
82. KF718668.1
83. KF718669.1
84. KF718670.1
85. KF718671.1
86. KF718673.1
87. KF718674.1
88. KF718675.1
89. KF718676.1
90. KF718677.1
91. KF718678.1
92. KF718679.1
93. KF718680.1
94. KU215404.1
95. KU215407.1
96. KU949329.1
97. KU949330.1
98. KU949331.1
99. KU949332.1
100. KU949333.1
101. KU949335.1
102. KU949336.1
103. KU949337.1
104. KU949338.1
105. KU949339.1
106. KU949340.1
107. KU949341.1
108. KU949343.1
109. KU949344.1
110. KU949345.1
111. KU949346.1
112. KU949347.1
113. KU949348.1
114. KU949349.1
115. KU949350.1
116. KU949351.1
117. KU949352.1
118. KY883330.1
119. KY883331.1
120. L28145.1
121. U31310.1
122. KU949334.1
PDV-F
PDV-P
PDV-R

PNRSV

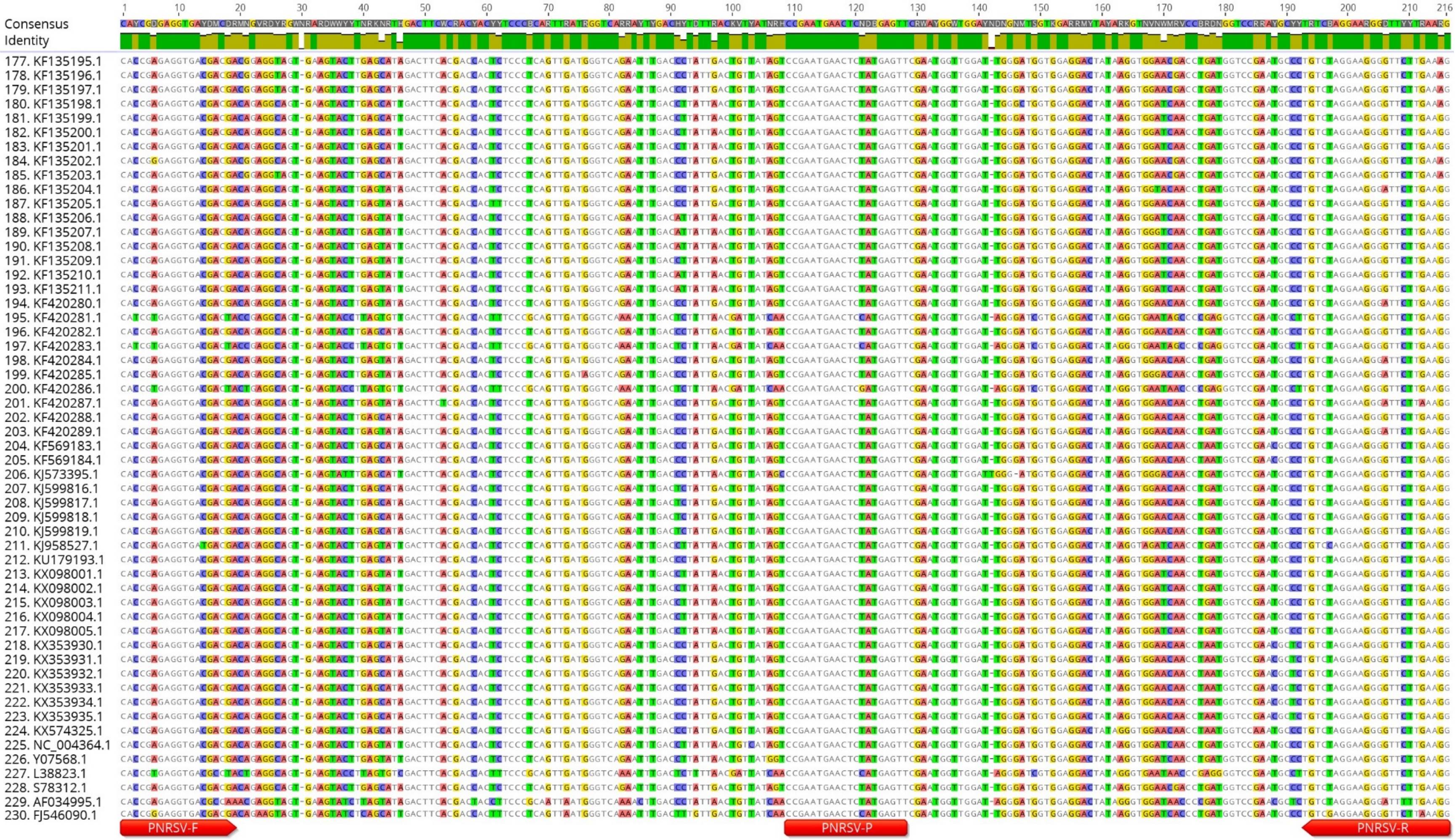

1 10 20 30 40 50 60 70 80 90 100 110 120 130 140 150 160 170 180 190 200 210 216
Consensus
Identity
177. KF135195.1
178. KF135196.1
179. KF135197.1
180. KF135198.1
181. KF135199.1
182. KF135200.1
183. KF135201.1
184. KF135202.1
185. KF135203.1
186. KF135204.1
187. KF135205.1
188. KF135206.1
189. KF135207.1
190. KF135208.1
191. KF135209.1
192. KF135210.1
193. KF135211.1
194. KF420280.1
195. KF420281.1
196. KF420282.1
197. KF420283.1
198. KF420284.1
199. KF420285.1
200. KF420286.1
201. KF420287.1
202. KF420288.1
203. KF420289.1
204. KF569183.1
205. KF569184.1
206. KJ573395.1
207. KJ599816.1
208. KJ599817.1
209. KJ599818.1
210. KJ599819.1
211. KJ958527.1
212. KU179193.1
213. KX098001.1
214. KX098002.1
215. KX098003.1
216. KX098004.1
217. KX098005.1
218. KX353930.1
219. KX353931.1
220. KX353932.1
221. KX353933.1
222. KX353934.1
223. KX353935.1
224. KX574325.1
225. NC_004364.1
226. Y07568.1
227. L38823.1
228. S78312.1
229. AF034995.1
230. FJ546090.1
PNRSV-F
PNRSV-P
PNRSV-R
