# Supplementary material for: Comprehensive Real-Time RT-PCR Assays for the Detection of Fifteen Viruses Infecting Prunus spp
Source: Plants (Basel). 2020 Feb 19;9(2):273. doi: 10.3390/plants9020273 (PMC7076543; doi:10.3390/plants9020273)
Supplement: Supplementary file 1 [file plants-09-00273-s001.zip › Figure S2.pdf]

Figure S2. Amplification plot and standard curve generated from new/updated real-time RT-PCR assays. In the plot, cycle threshold (C_T) values obtained for three replicates of ten-fold serial dilutions of *Prunus* samples infected by different viruses. Eff%, amplification efficiency in percentage; R^2, coefficient of correlation.

ACLSV

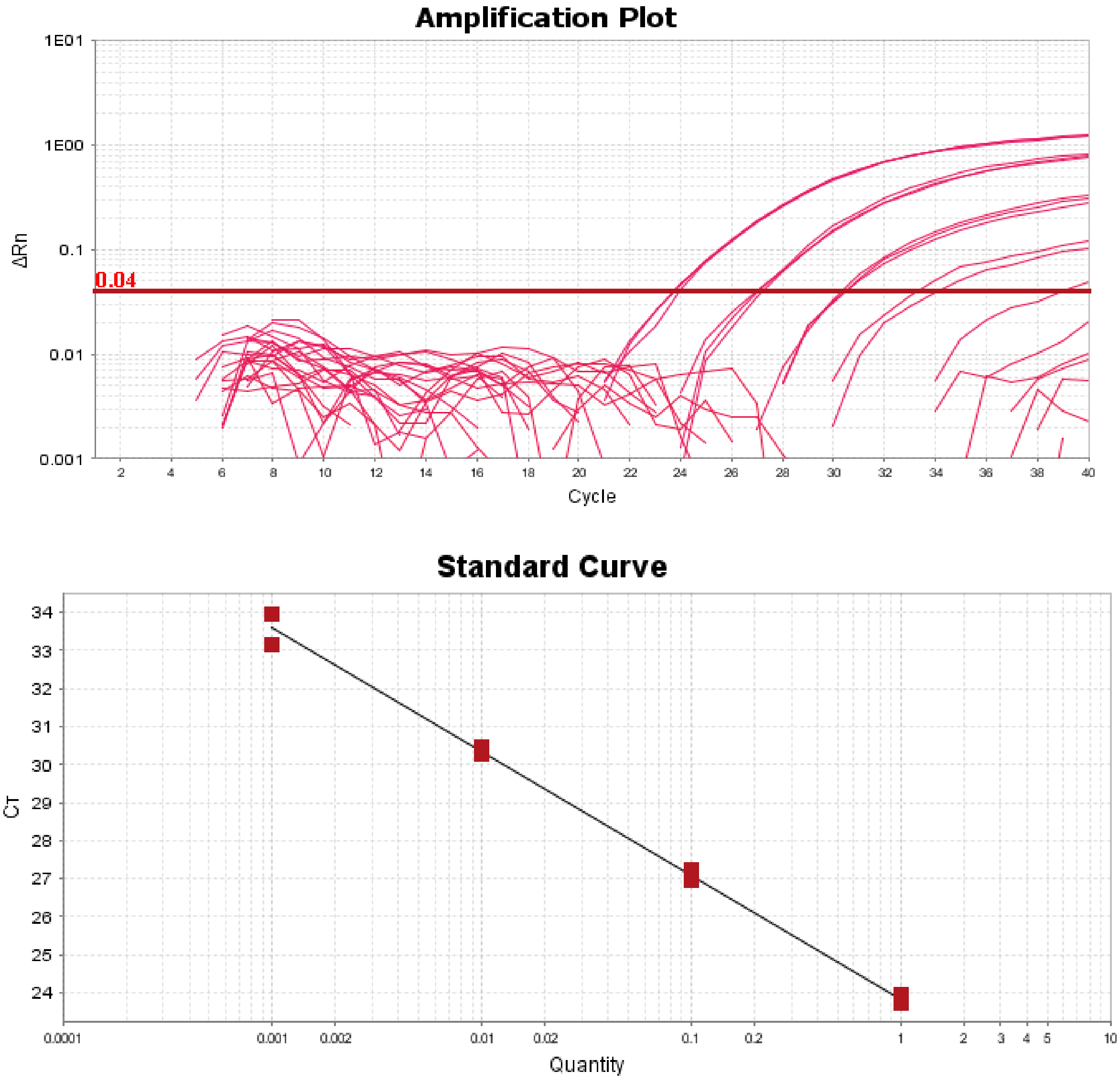

Slope: -3.26
R^2: 0.997
Eff%: 102.66
Error: 0.061

CGRMV

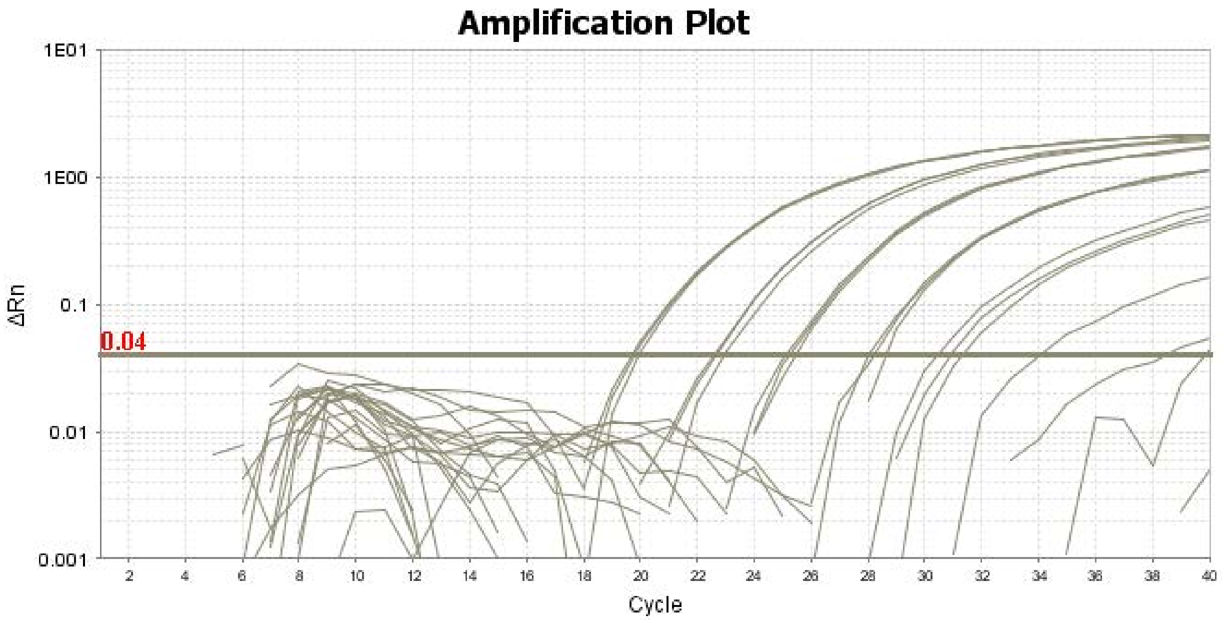

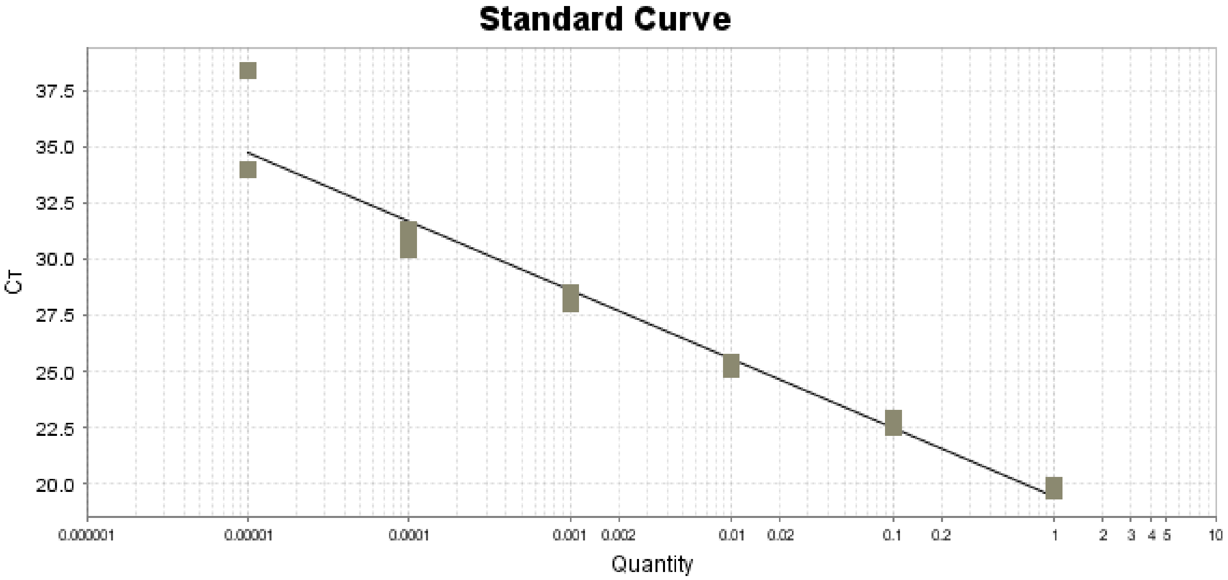

Slope: -3.056
R^2: 0.959
Eff%: 112.44
Error: 0.163

CLRV

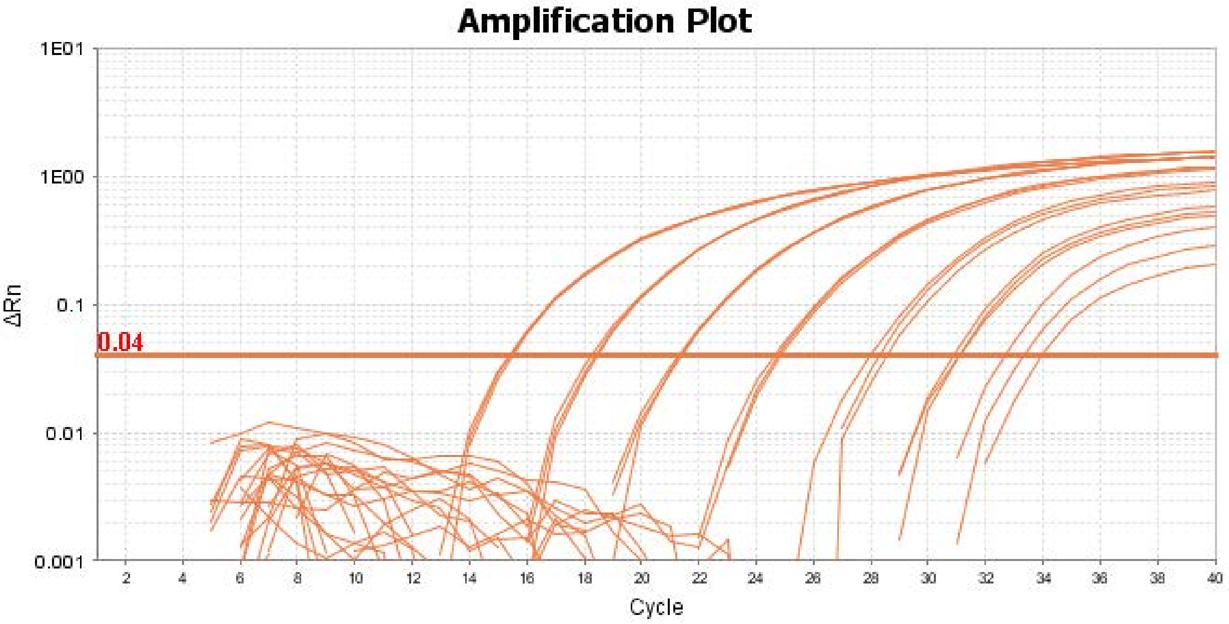

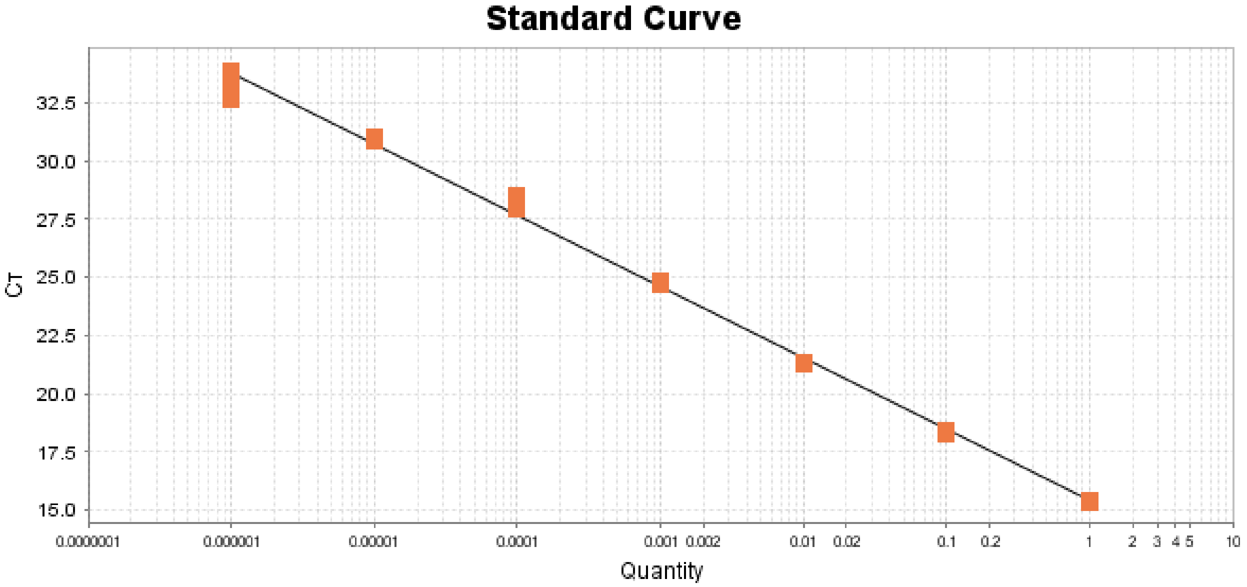

Slope: -3.065
R^2: 0.996
Eff%: 111.955
Error: 0.046

CNRMV

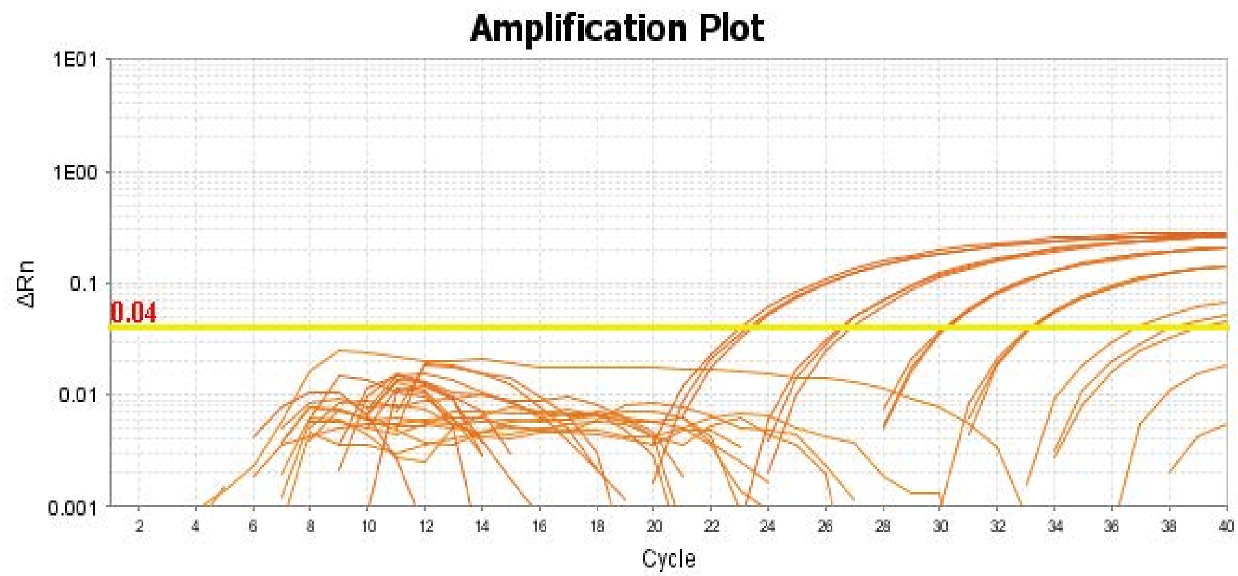

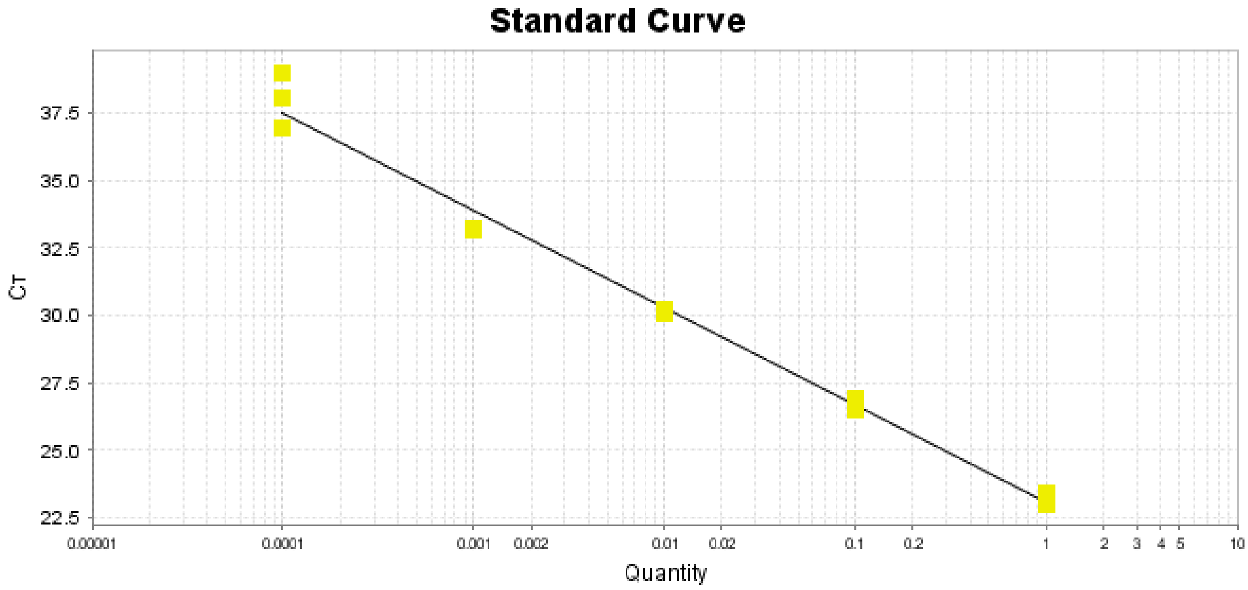

Slope: -3.608
R^2: 0.988
Eff%: 89.322
Error: 0.11

CRLV

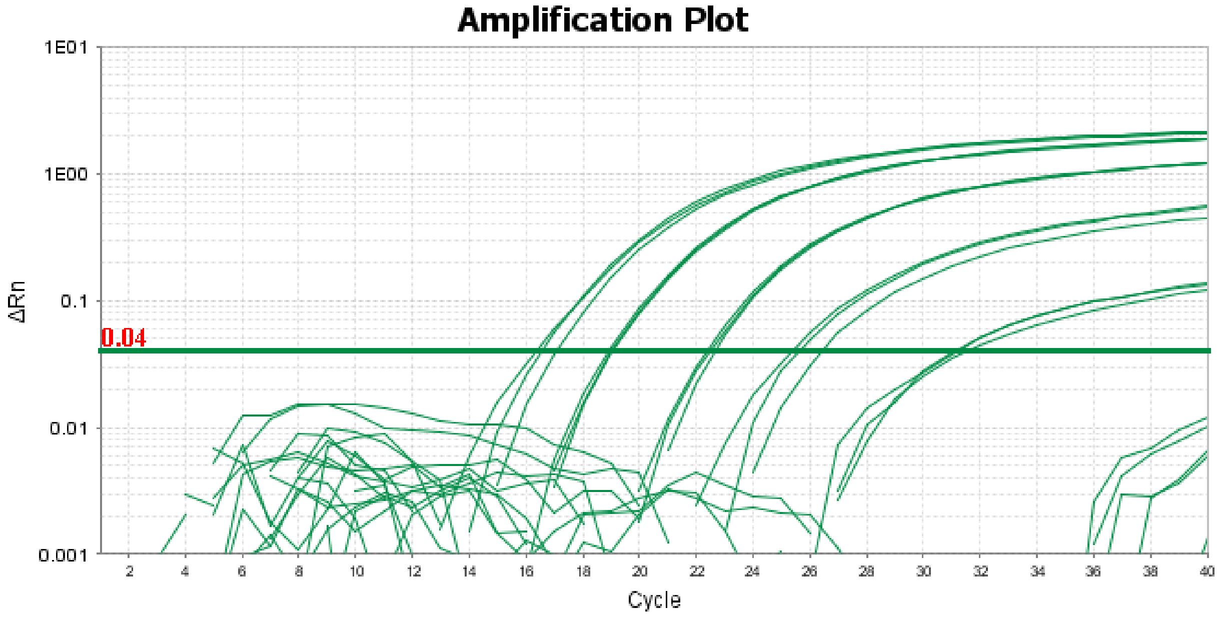

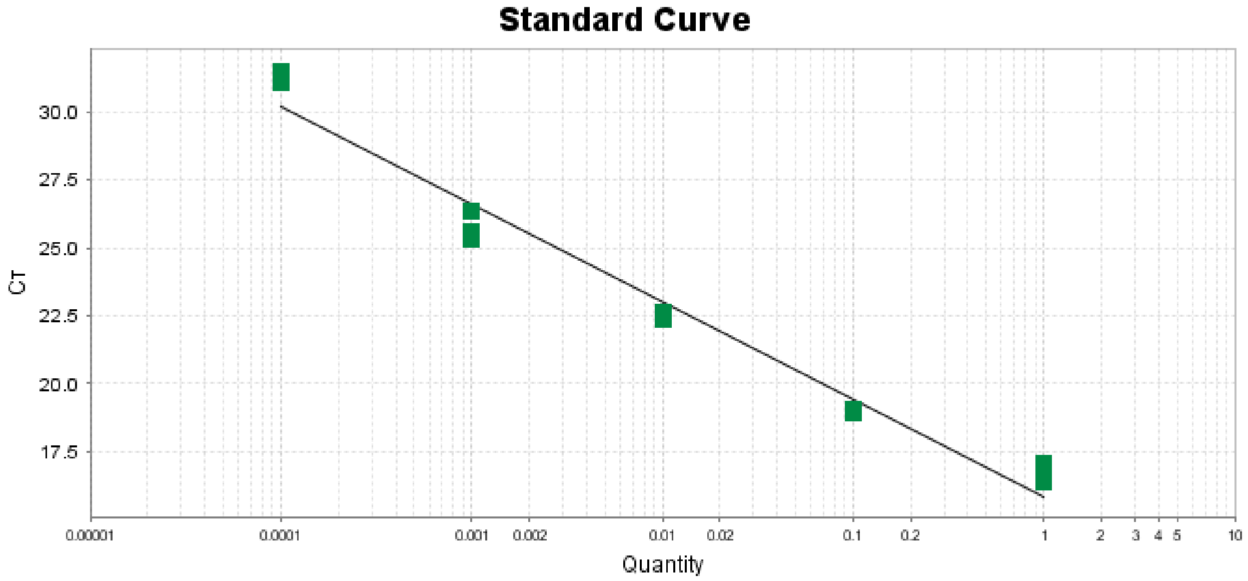

Slope: -3.597
R^2: 0.975
Eff%: 89.685
Error: 0.16

CRMaV

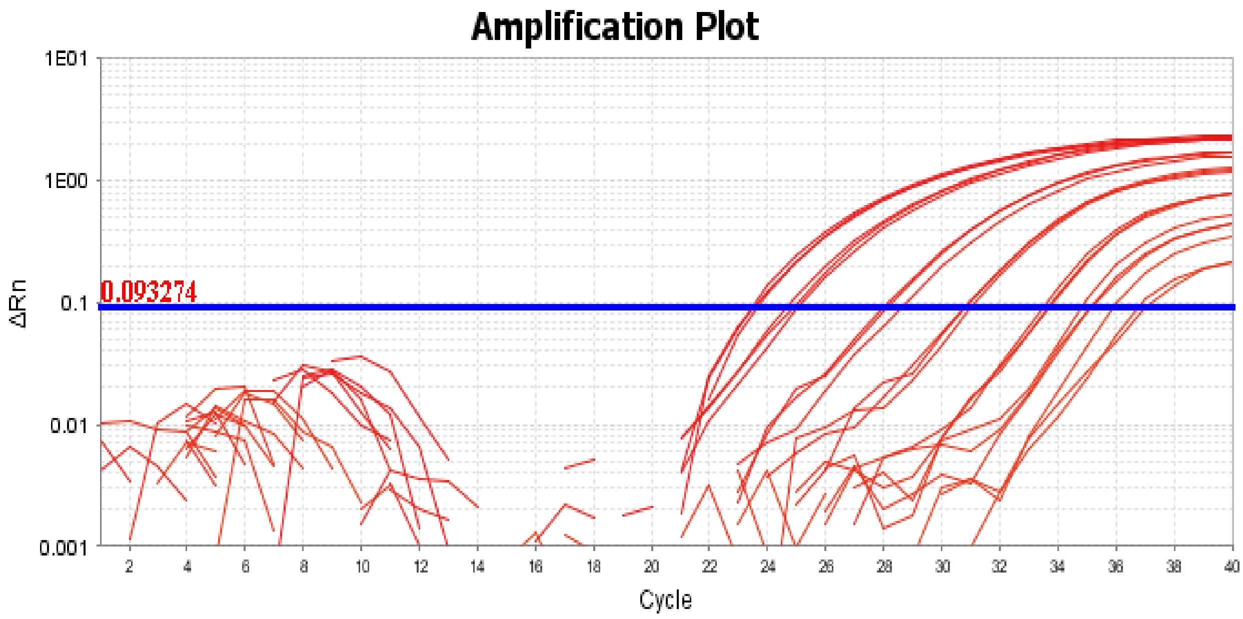

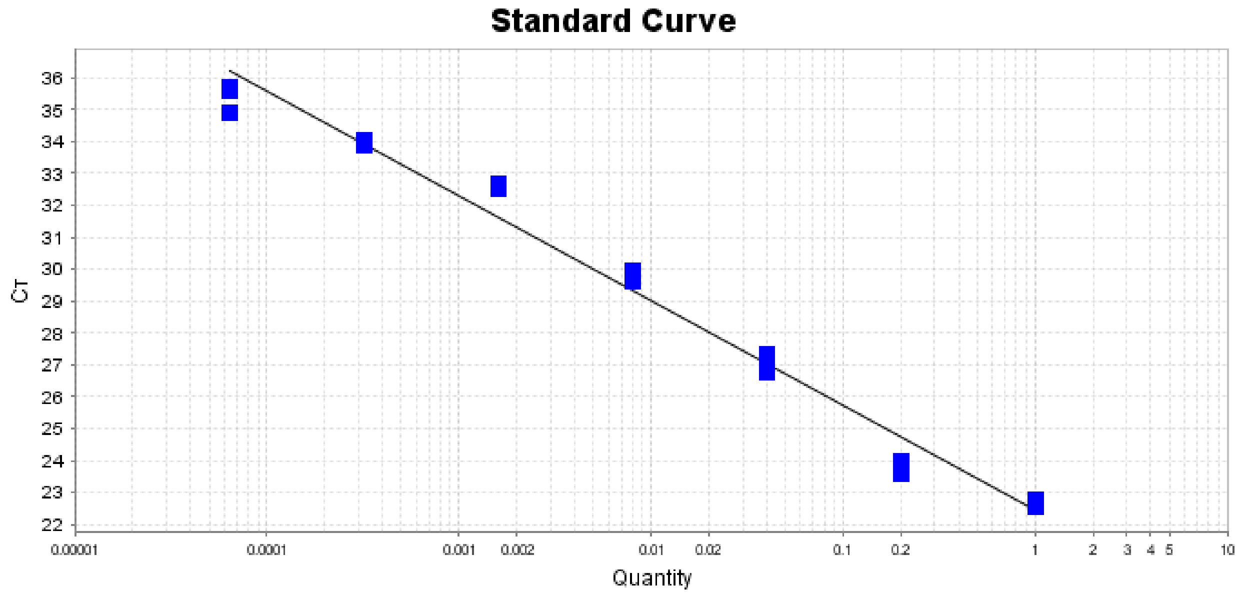

Slope: -3.285
R^2: 0.98
Eff%: 101.564
Error: 0.108

CVA

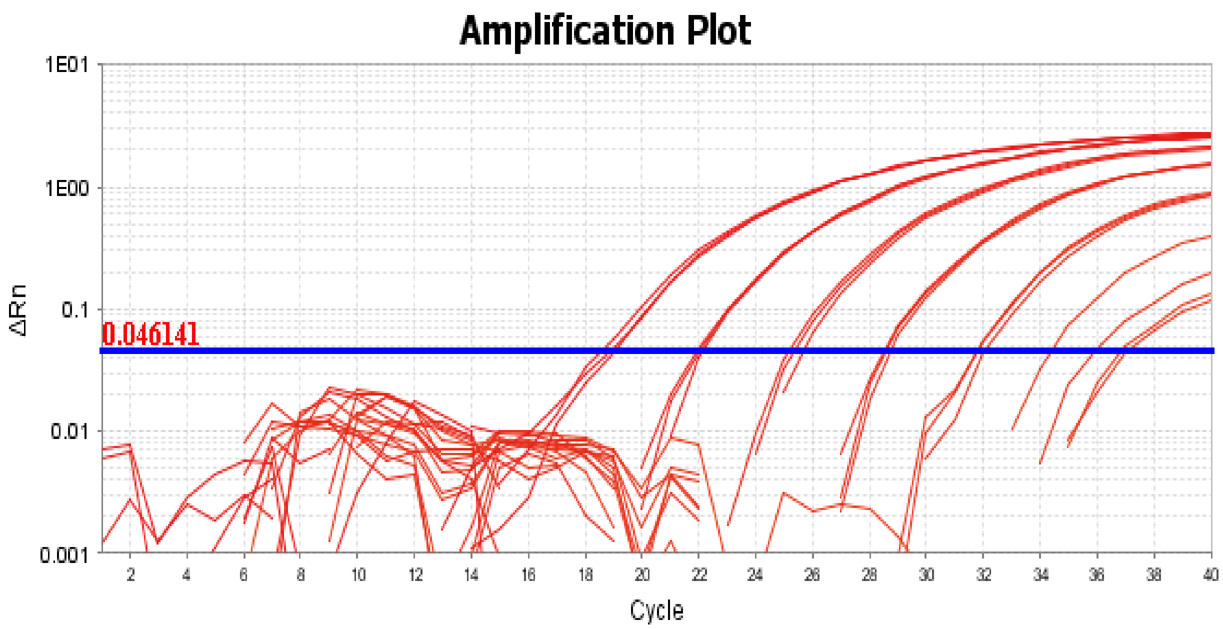

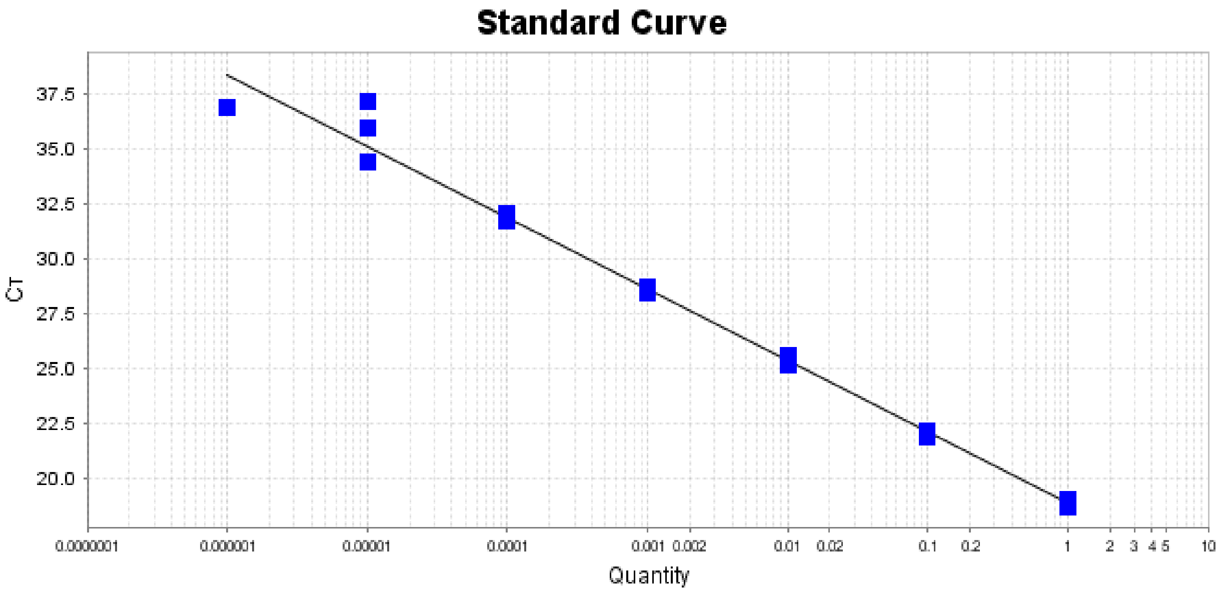

Slope: -3.253
R^2: 0.989
Eff%: 102.946
Error: 0.085

LChV-1

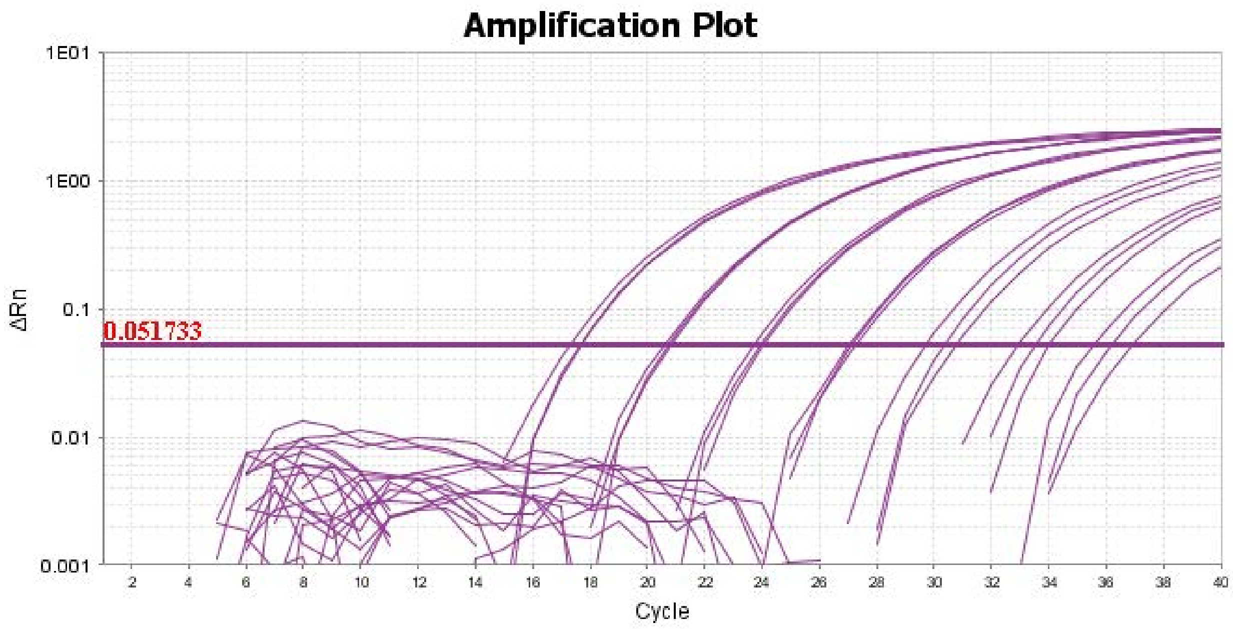

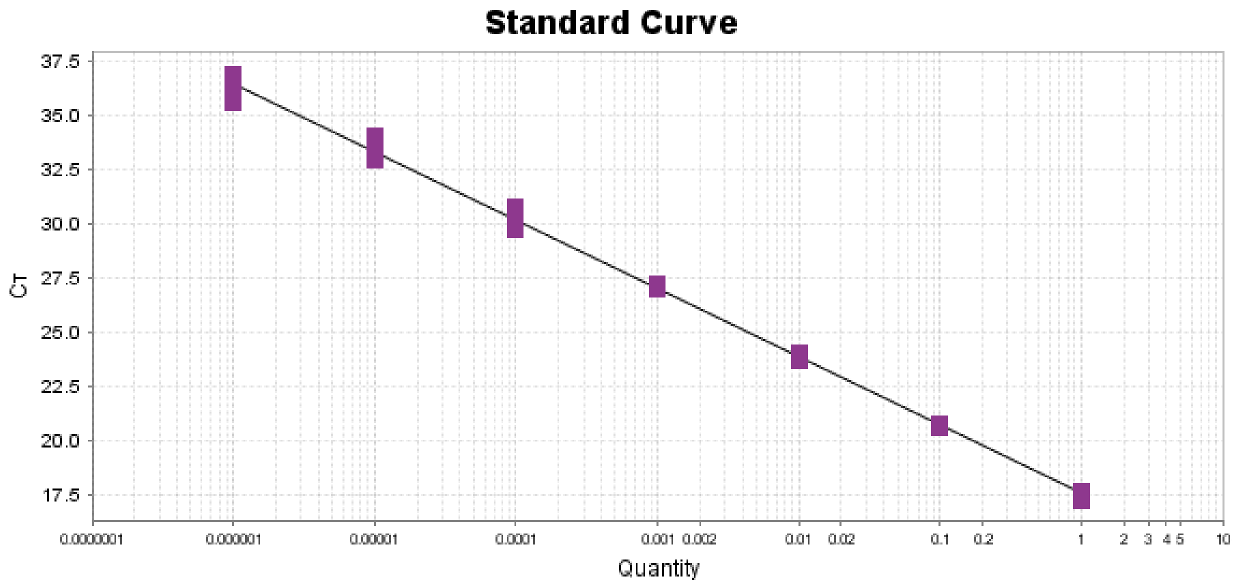

Slope: -3.145
R^2: 0.997
Eff%: 107.948
Error: 0.04

LChV-2

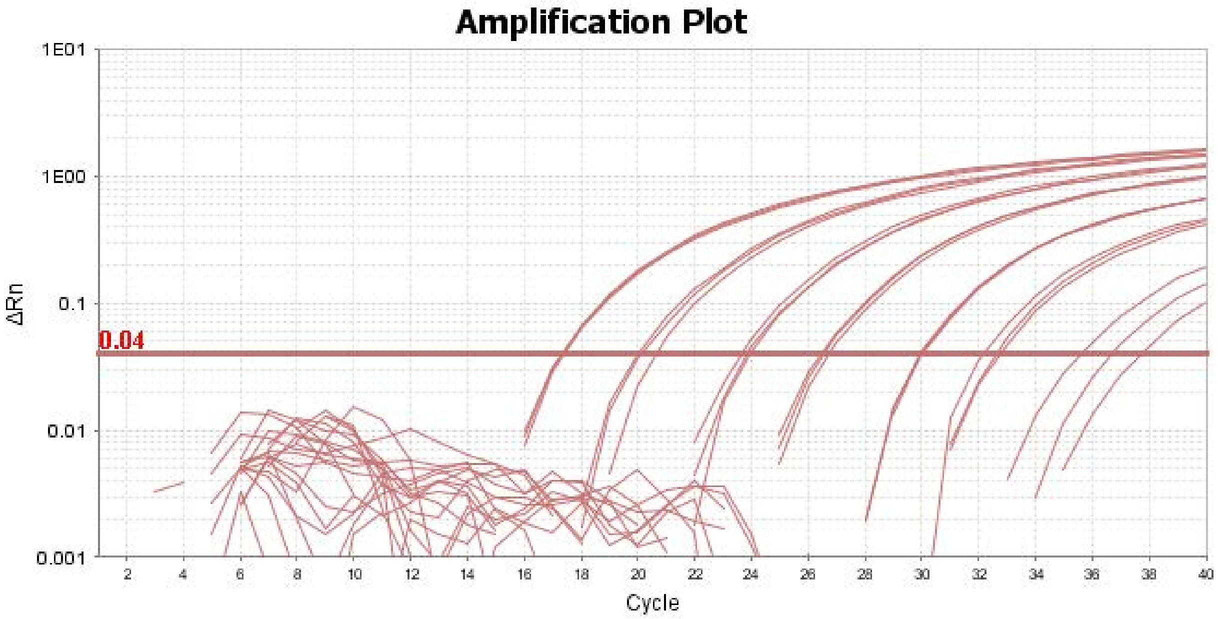

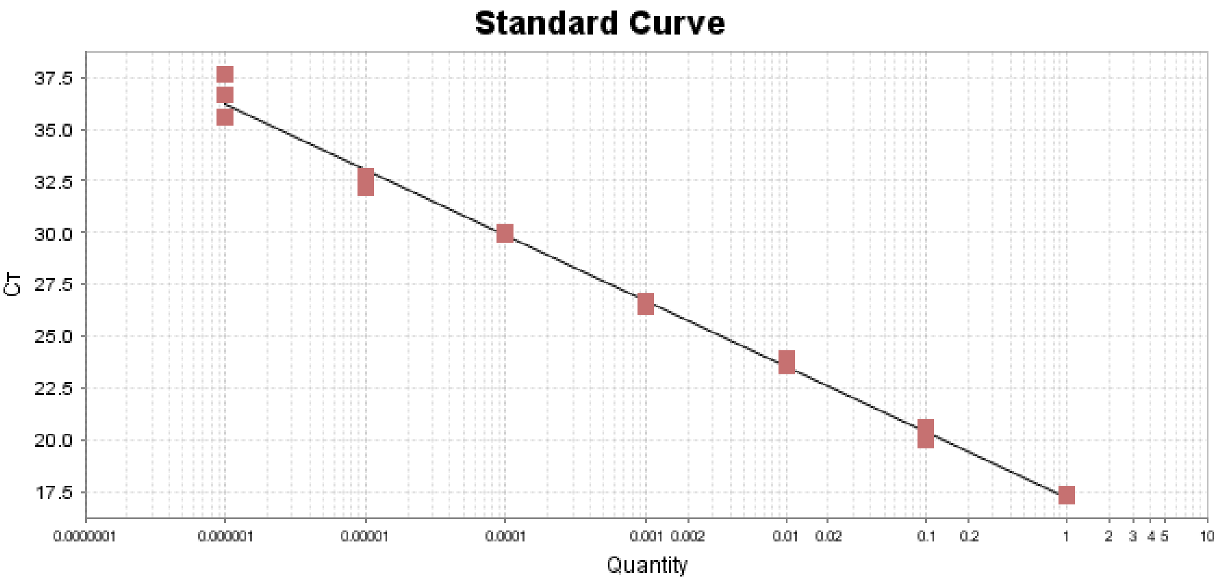

Slope: -3.17
R^2: 0.995
Eff%: 106.756
Error: 0.059

NSPaV

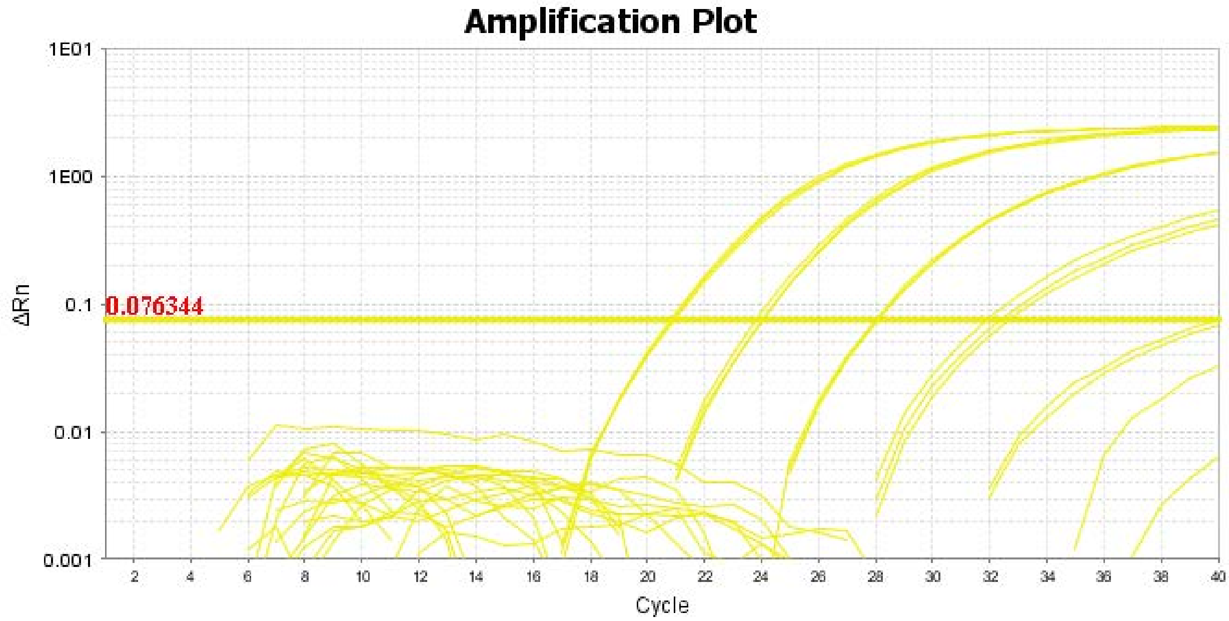

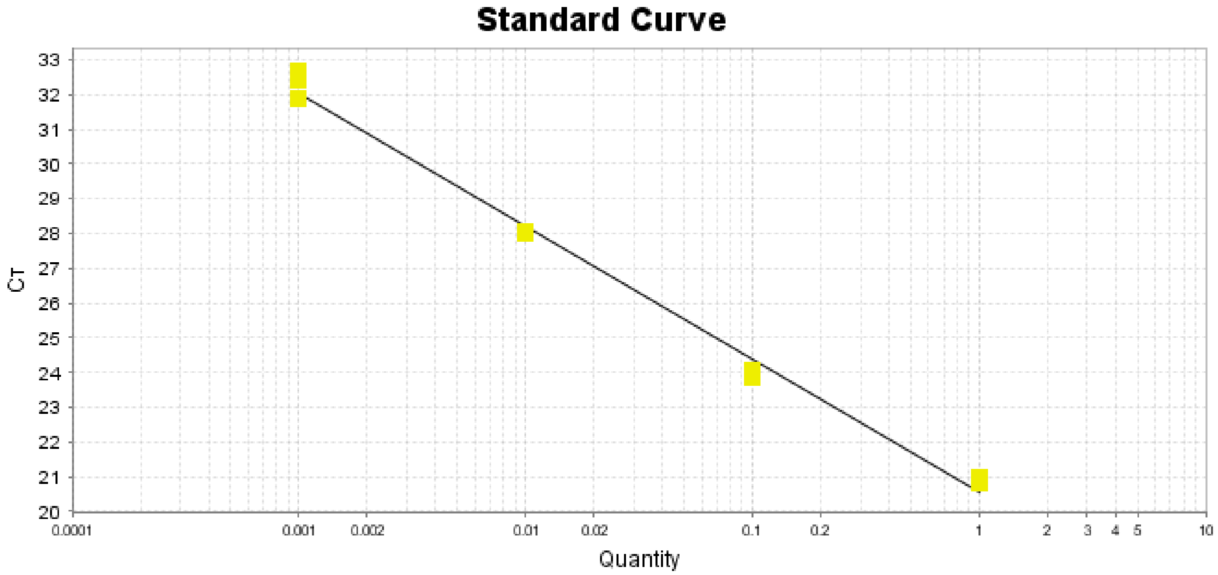

Slope: -3.835
R^2: 0.993
Eff%: 82.29
Error: 0.103

NVM

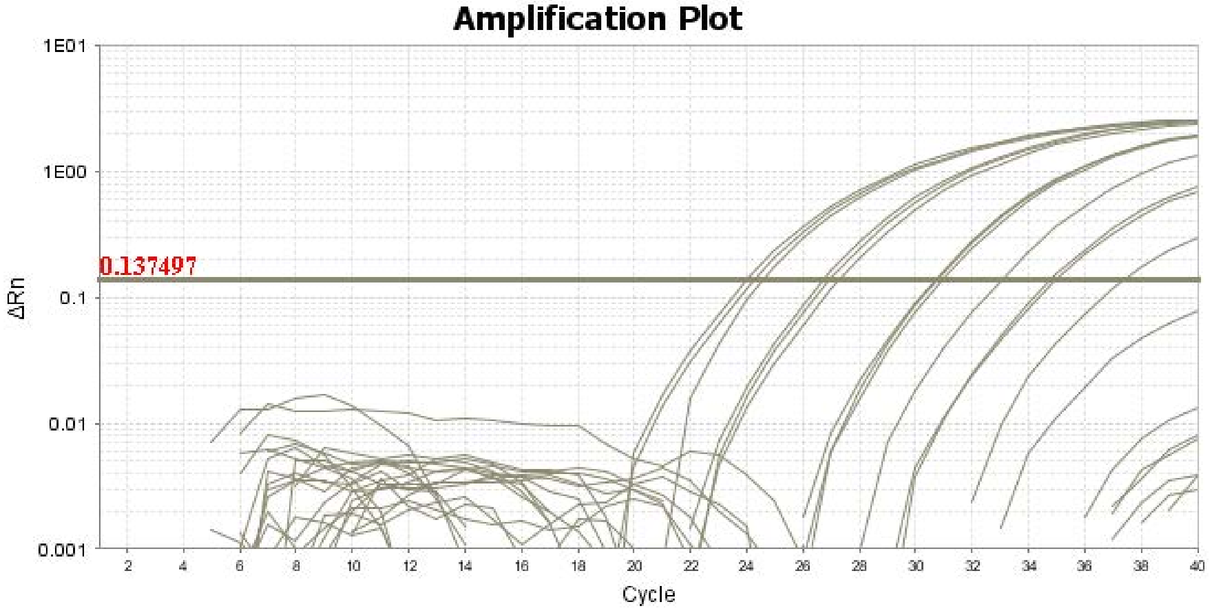

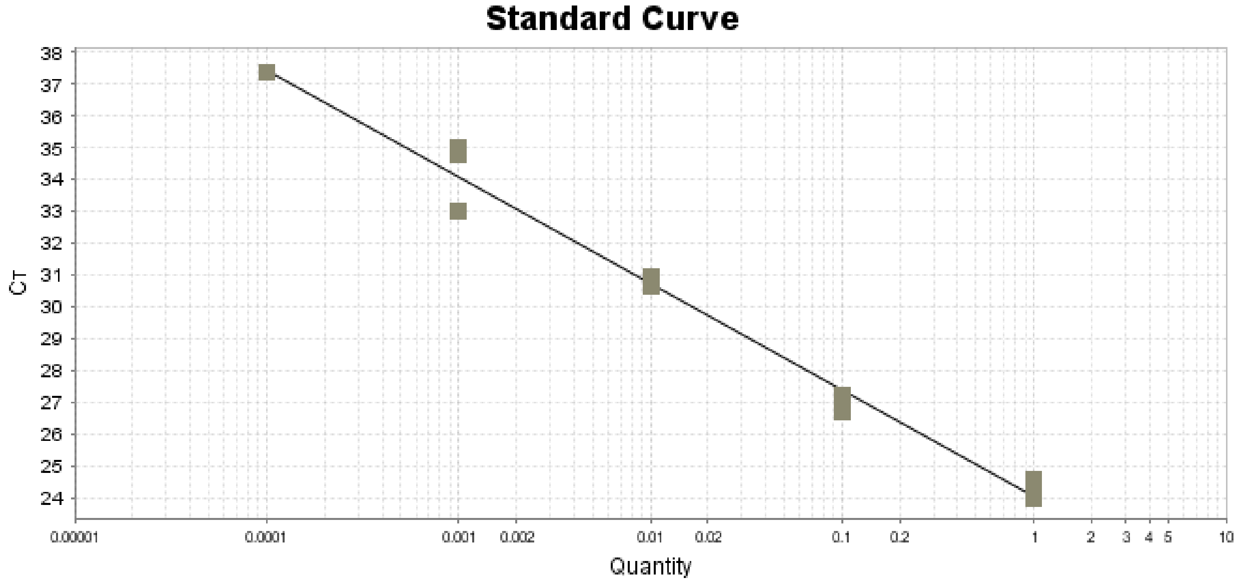

Slope: -3.363
R^2: 0.985
Eff%: 98.314
Error: 0.124

PBNSPaV

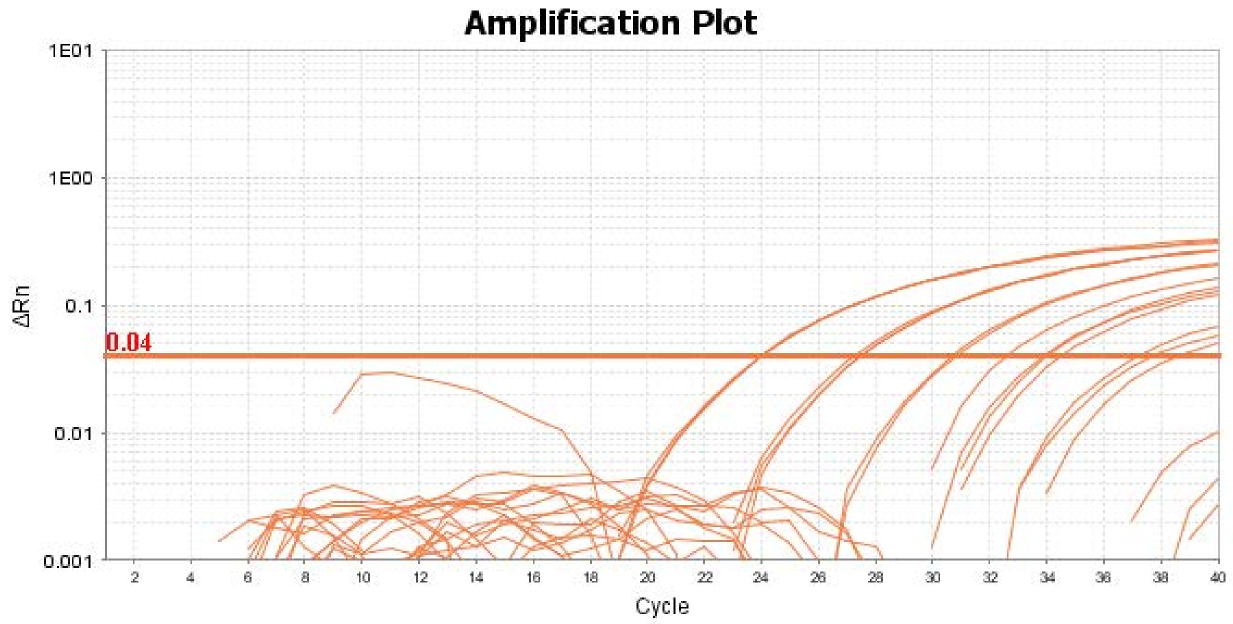

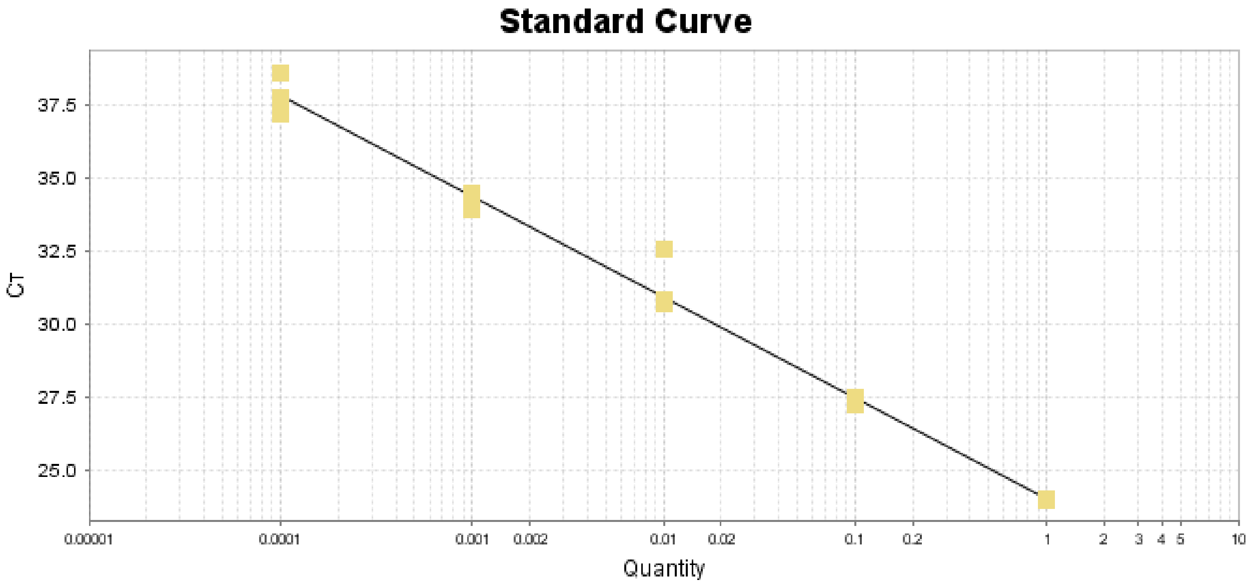

Slope: -3.447
R^2: 0.988
Eff%: 95.036
Error: 0.104

PcMV

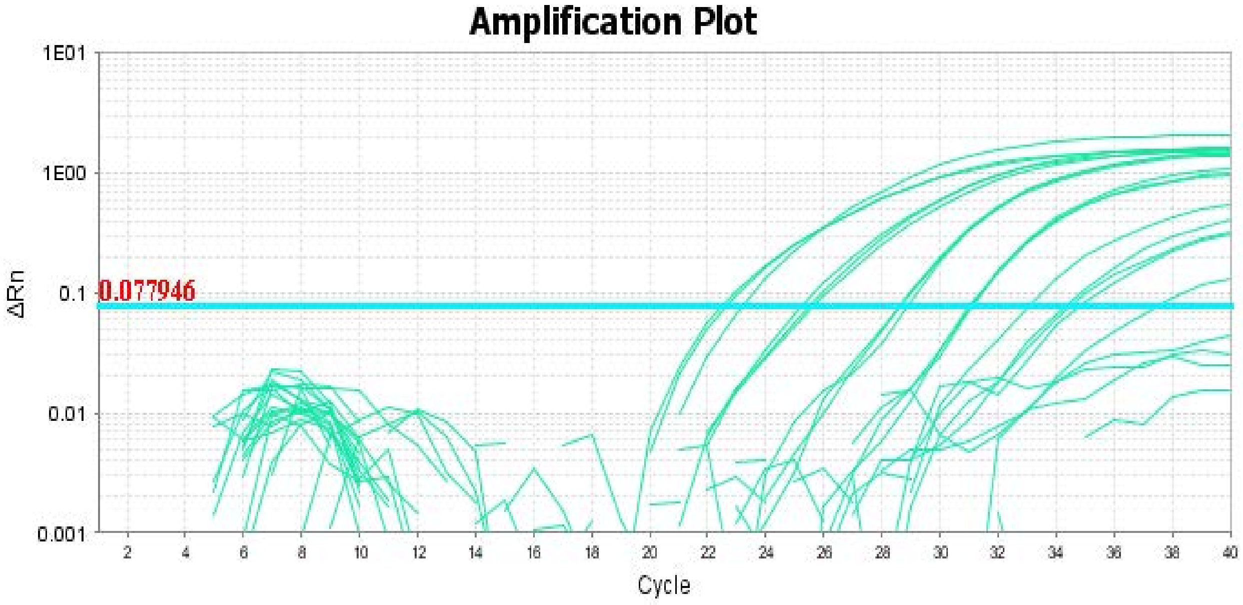

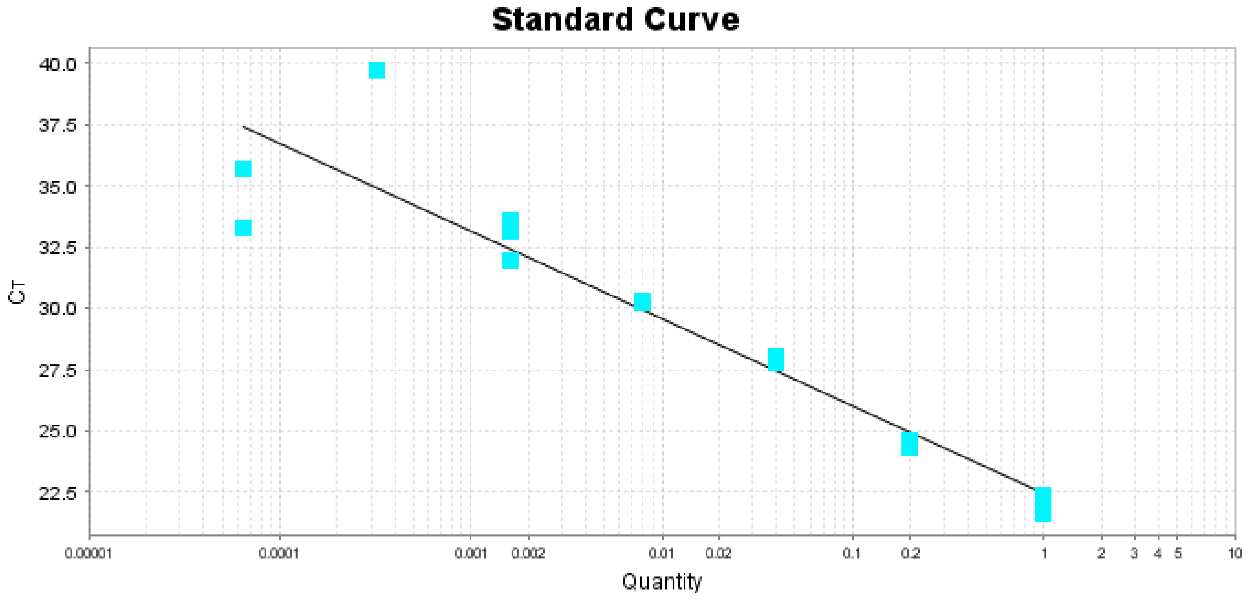

Slope: -3.558
R^2: 0.892
Eff%: 91.021
Error: 0.31

PDV

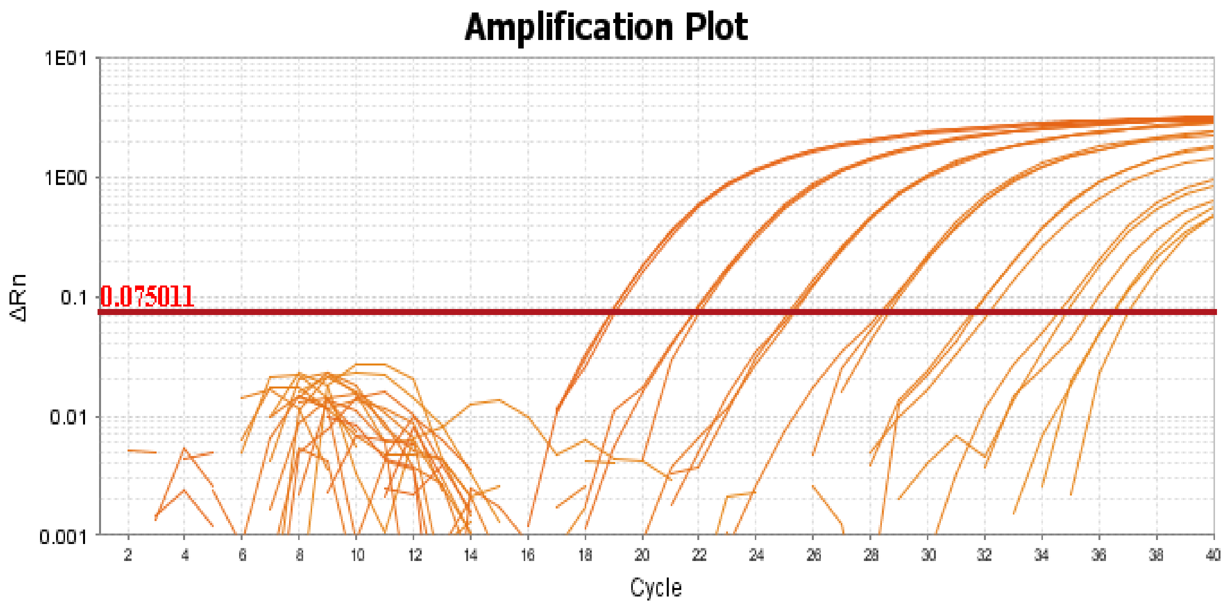

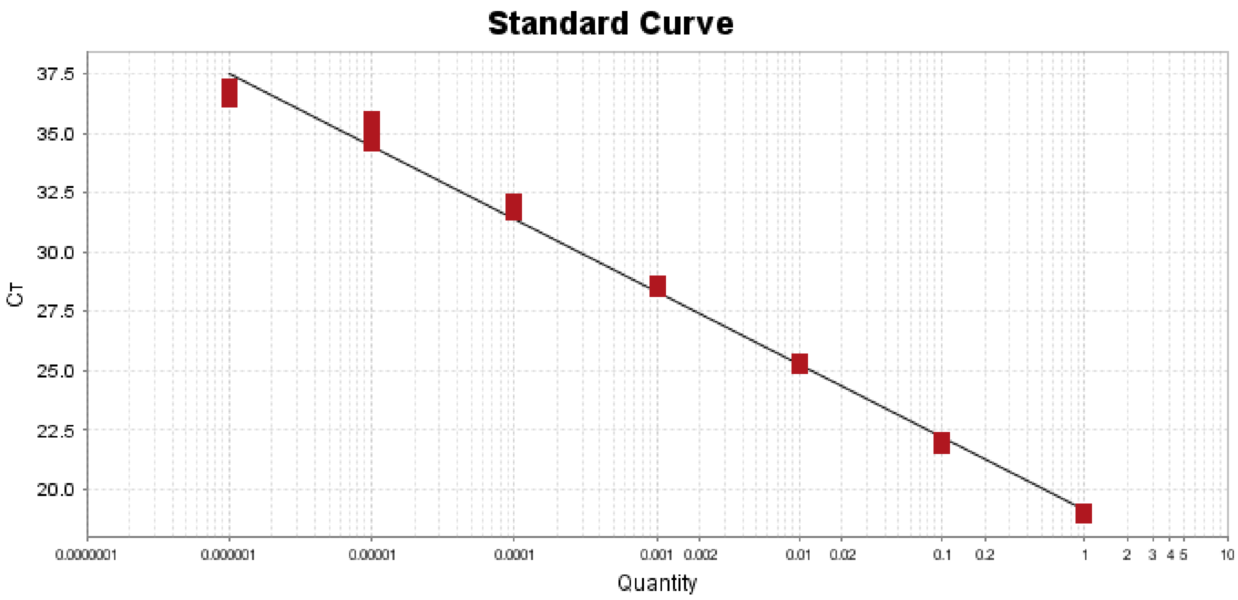

Slope: -3.06
R^2: 0.993
Eff%: 112.22
Error: 0.058

PNRSV

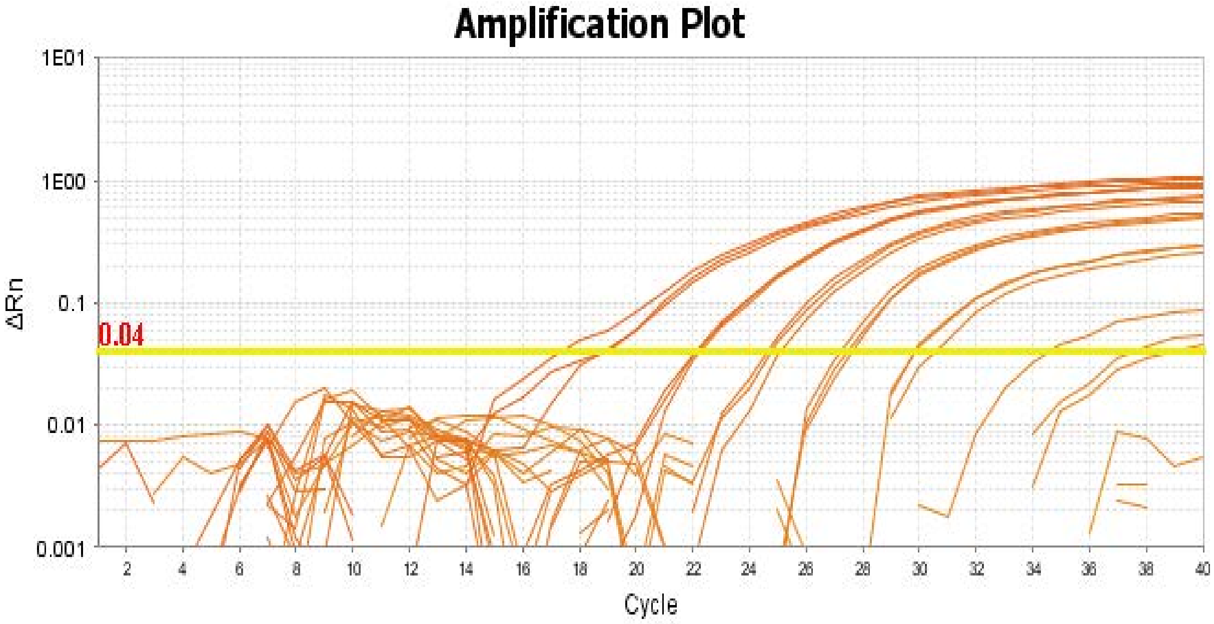

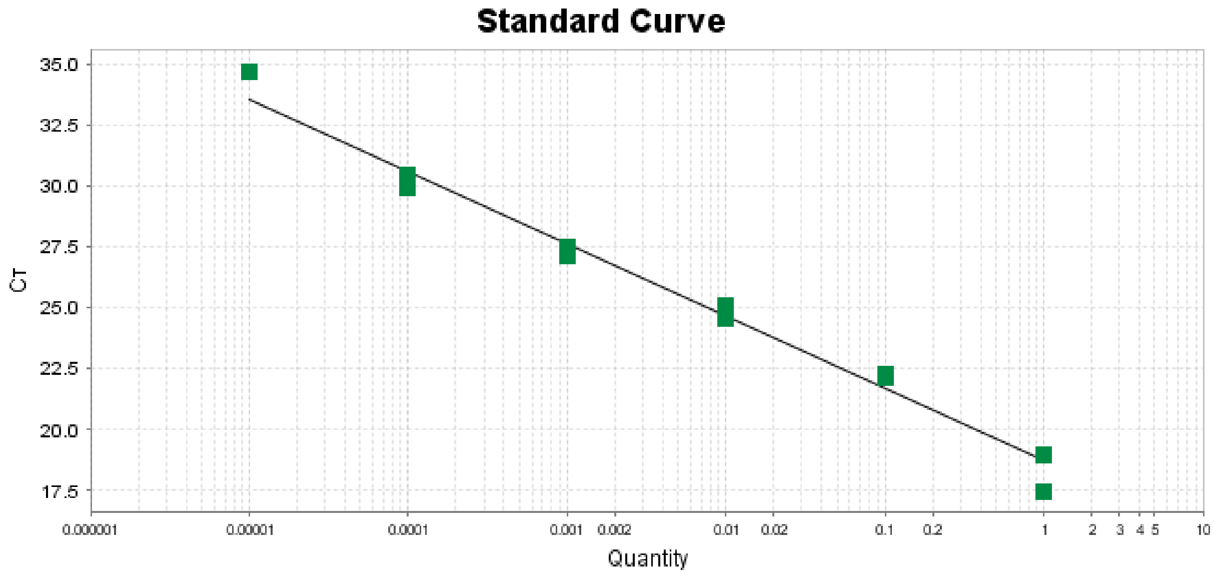

Slope: -2.965
R^2: 0.985
Eff%: 117.382
Error: 0.098
